# Incorporating Motif Analysis into Gene Co-expression Networks Reveals Novel Modular Expression Pattern and New Signaling Pathways

**DOI:** 10.1371/journal.pgen.1003840

**Published:** 2013-10-03

**Authors:** Shisong Ma, Smit Shah, Hans J. Bohnert, Michael Snyder, Savithramma P. Dinesh-Kumar

**Affiliations:** 1 Department of Plant Biology and the Genome Center, College of Biological Sciences, University of California, Davis, Davis, California, United States of America; 2 Departments of Plant Biology and Crop Sciences, University of Illinois at Urbana-Champaign, Urbana, Illinois, United States of America, and Division of Life Sciences, Gyeongsang National University, Jinju, Korea; 3 Department of Genetics, Stanford University, Stanford, California, United States of America; The University of North Carolina at Chapel Hill, United States of America

## Abstract

Understanding of gene regulatory networks requires discovery of expression modules within gene co-expression networks and identification of promoter motifs and corresponding transcription factors that regulate their expression. A commonly used method for this purpose is a top-down approach based on clustering the network into a range of densely connected segments, treating these segments as expression modules, and extracting promoter motifs from these modules. Here, we describe a novel bottom-up approach to identify gene expression modules driven by known *cis*-regulatory motifs in the gene promoters. For a specific motif, genes in the co-expression network are ranked according to their probability of belonging to an expression module regulated by that motif. The ranking is conducted *via* motif enrichment or motif position bias analysis. Our results indicate that motif position bias analysis is an effective tool for genome-wide motif analysis. Sub-networks containing the top ranked genes are extracted and analyzed for inherent gene expression modules. This approach identified novel expression modules for the G-box, W-box, site II, and MYB motifs from an *Arabidopsis thaliana* gene co-expression network based on the graphical Gaussian model. The novel expression modules include those involved in house-keeping functions, primary and secondary metabolism, and abiotic and biotic stress responses. In addition to confirmation of previously described modules, we identified modules that include new signaling pathways. To associate transcription factors that regulate genes in these co-expression modules, we developed a novel reporter system. Using this approach, we evaluated MYB transcription factor-promoter interactions within MYB motif modules.

## Author Summary

Gene co-expression networks unite genes with similar expression patterns. From these networks, gene co-expression modules can be identified. A specific family of transcription factor(s) may regulate the genes within a co-expression module. Thus, module identification is important to decipher the gene regulatory network. Previously, module identification relied on clustering the gene network into gene clusters that were then treated as modules. This represents a top-down approach. Here, we introduce a reverse approach aiming at identifying gene co-expression modules regulated by known promoter motifs. For a given promoter motif, we calculated the probability of each gene within the network to belong to a module regulated by that motif *via* motif enrichment analysis or motif position bias analysis. A sub-network containing the genes with a high probability of belonging to a motif driven module was then extracted from the gene co-expression network. From this sub-network, the modular structure can be identified *via* visual inspection. Our bottom-up approach recovered many known and novel modules for the G-box, MYB, W-box and site II elements motif, whose expression may be regulated by the transcription factors that bind to these motifs. Additionally, we developed a rapid transcription factor-promoter interaction screening system to validate predicted interactions.

## Introduction

The advancement in technologies in recent years has resulted in many large data sets cataloging the biological systems at various levels. Biological networks inferred from these data have become an important tool to describe and analyze biological signaling systems [1]–[3]. Depending on the sources of the data, different biological networks include information on protein-protein and protein-DNA interactions, or network structures for gene co-expression, metabolism, phosphorylation, and yet other structured sets that integrate diverse data sources. Identifying novel signaling or gene expression modules from these networks has become a major goal of systems biology.

Plant biological networks are mainly gene co-expression networks based on large-scale transcriptome data. Relatively few studies on protein-protein interaction [1], [4], [5], protein-DNA interaction [6], [7] or phosphorylation [8] have been reported. The gene co-expression networks consist of nodes representing genes and edges representing connections between nodes. An edge between two genes indicates that they have similar expression patterns under various biological conditions. The pair-wise gene expression similarities are mostly measured using the Pearson correlation coefficient [9]–[12]. In addition, association measurements have also been derived using Mutual Rank [13], the Spearman correlation coefficient [14], and the partial correlation coefficient [15]–[17] methods. Plant functional networks integrating multiple data types, including co-expression, have also been reported [18]–[21].

Once generated, these co-expression networks are used to identify expression modules to extract biological meaning. An expression module includes a subset of genes from within the network that are highly interconnected with each other but show only limited connection to genes outside the subset. Expression modules usually represent groups of co-expressed genes with condition-specific similar or same expression patterns, suggesting that they likely belong to gene expression units regulated by the same transcription factor(s) (TF). Various network clustering methods have been used to identify such modules from plant gene co-expression networks. These include Markov chain clustering (MCL) [9], [10], [22], [23], IPCA [12], NeMo algorithm [24], and HQcut [25]. In these methods the clustering algorithms while searching for modules only consider the topology and connectivity of the networks but fail to take into account the properties of the nodes or the genes such as promoter sequences. Motifs in the promoters are only searched after the modules are extracted. This represents a top-down strategy.

Here, we describe a bottom-up approach to identify expression modules from a previously published *Arabidopsis thaliana* gene co-expression network based on the graphical Gaussian model [15], [26]. Our major interest is to understand how known promoter motifs are distributed across the gene network and to identify gene expression modules that these motifs might regulate. For any given motif, every gene in the network was first analyzed to calculate its probability of belonging to an expression module regulated by that motif. Then, all the top ranked genes were used to extract a sub-network from the original gene co-expression network. From this sub-network, the modular structures will self-manifest, thus enabling discovery of novel signaling pathways. We used this approach to successfully identify novel expression modules for four well studied motifs - G-box, MYB, W-box, and site II element. We validated our predicted promoter-motif interactions using a novel *in vivo* reporter assay system. The bioinformatics program described here can be used to extract expression modules for any motif of interest.

## Results

### Network-based motif analysis

**Figure 1:**
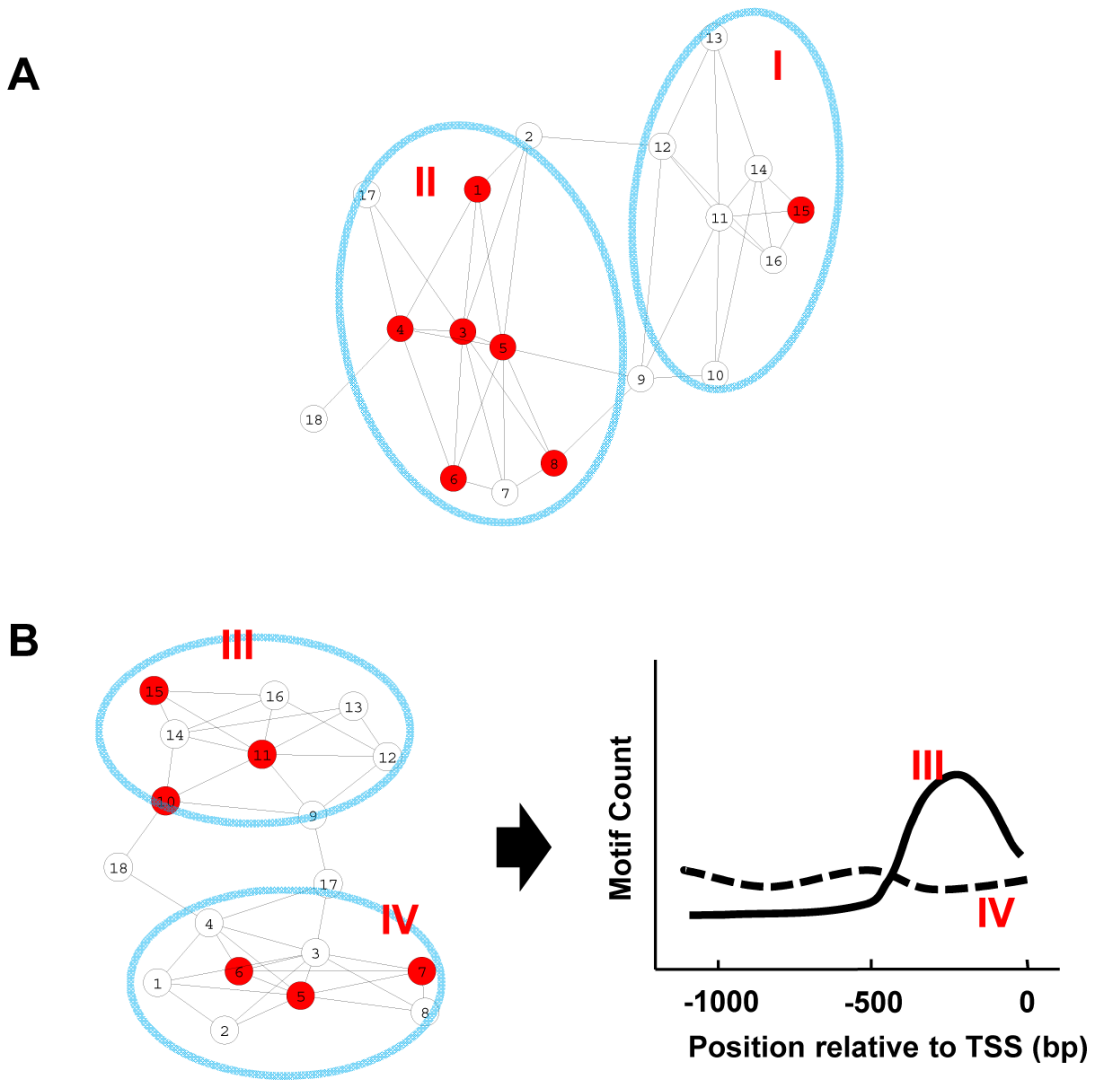
Motif enrichment and motif position bias analysis. (A) A representation of the co-expression network. Each node represents a gene, while a connection between two genes indicates similar expression pattern. Genes containing a given motif are shown in red. Genes in group II are enriched for this motif and are more likely to be regulated by that motif. (B) In this sub-network, both gene groups include similar numbers of genes containing the motif, but the motif positions distribute differently along the promoters in two modules. Group III genes display position bias for the motif and are more likely to be regulated by that motif.

Gene co-expression networks describe the pattern of co-expression between genes. The connected gene pairs within such networks share similar expression patterns. A subset of genes within such a network might be combined by the presence of a specific motif in their promoters. Depending on their expression patterns, some of these same-motif-containing genes cluster together and form expression modules, while others disperse across the network (Figure 1A). The genes in the former category cluster together at a frequency higher than random distribution. It is assumed that these clustered same-motif-containing genes belong to expression module(s) that will be regulated by the corresponding motif in a condition-specific manner. It is also important to note that the promoter motifs tend to show position bias in their distribution relative to the transcription start site (TSS). Consider two groups of genes containing the same motif in their promoters with similar frequency. We can distinguish them by one where a motif is distributed evenly along the promoters and the other where the motif is skewed towards being present closer to the TSS (Figure 1B). The probability for the latter group of genes to be regulated by that motif is higher than the former group.

Thus, by studying how a specific motif distributes across the network, it is possible to identify the expression modules it regulates. The key is to distinguish the same-motif-containing genes belonging to expression modules with motif enrichment/motif position bias from those that do not belong. For this, we employed two independent methods. One is based on the hypergeometric distribution to assess motif enrichment and the other is based on the uniform distribution to measure motif position bias towards TSS. Specifically for each motif, a pValue of motif enrichment and a z-score for motif position bias were calculated for every gene within the network in the following manner. For any gene, the gene and its immediately connected neighbor genes within the network are considered as a group. The frequency of the motif present within the promoters of this group of genes is compared to those of the whole genome and a pValue based on the hypergeometric distribution is calculated. The locations of the motif within these promoters are also used to compute a z-score as an indicator of whether the motif has a position-bias distribution towards TSS as described before [27] (see Material and Methods). A large z-score indicates the motif has a biased distribution towards TSS, while a motif with even distribution along the promoters will result in a z-score close to zero.

Genes are then ranked according to their pValues. The smaller the pValue the higher the chance that the gene belongs to an expression module regulated by the motif that is under consideration. All genes with pValues smaller than a selected cut-off are used as seeds to generate a sub-network from the original co-expression network. The sub-network is inspected for the existence of densely connected modules that provide information about the propensity of the motif to drive the modular expression of its targets. As an independent method, genes are also ranked according to their z-score. The genes with z-scores larger than a selected cut-off are extracted and used to generate sub-networks. The sub-networks are then inspected for module structures.

For our analysis, we used an Arabidopsis gene co-expression network that had been established based on the graphical Gaussian model (GGM) [15], [26]. With a partial correlation co-efficient cut off at 0.05 [26], it contains 16,459 genes (nodes) and 120,276 co-expressed gene pairs (edges) (Table S1). Here, we focused our analysis on the 10,385 nuclear-encoded genes connected to 5 or more co-expressed genes, i.e. nodes with > = 5 edges.

### Expression modules regulated by the G-box motif in a co-expression network

**Figure 2:**
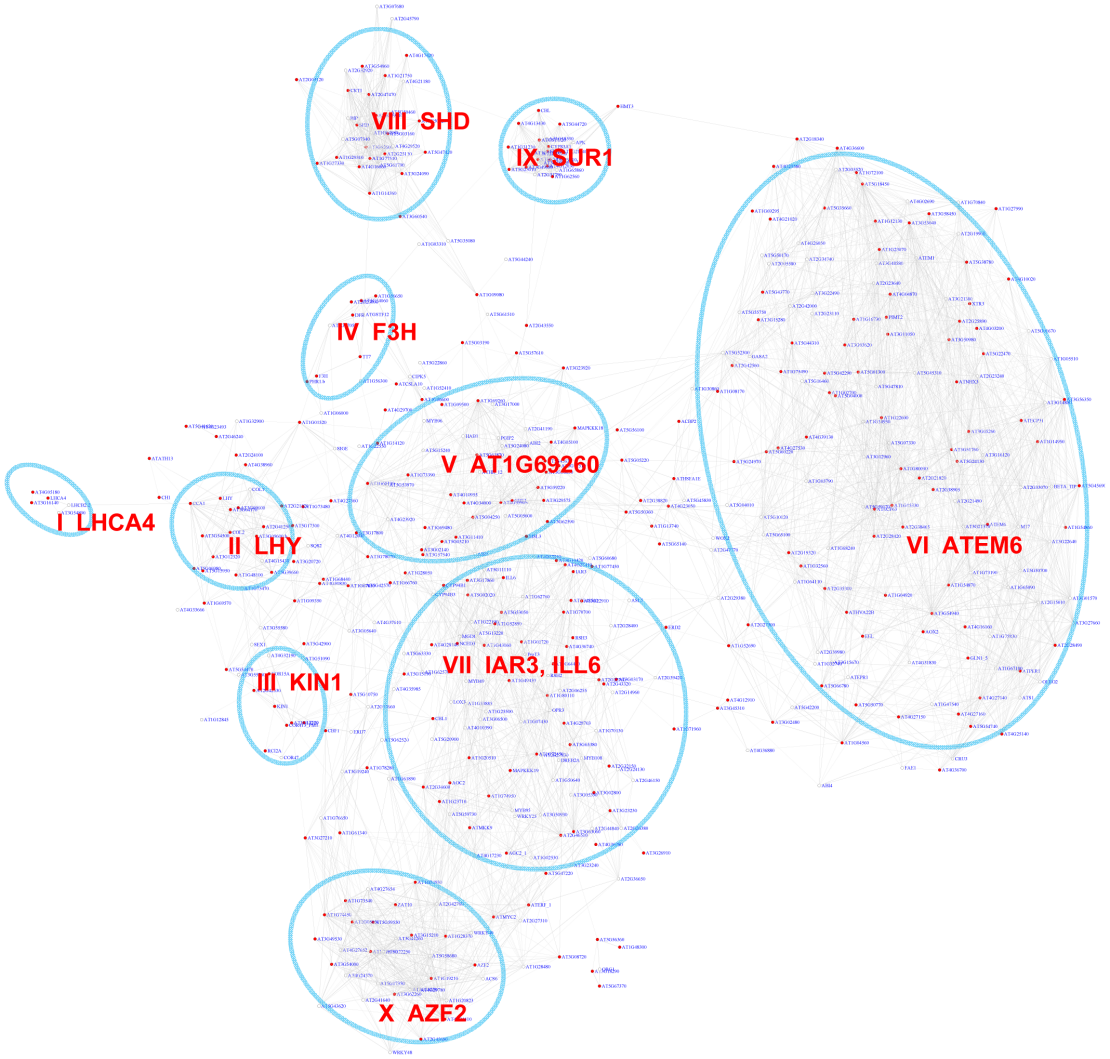
A sub-network for the G-box motif based on motif enrichment analysis. Genes were identified by motif enrichment analysis with pValue for the G-box motif < = 0.001. Eleven modules were identified and labeled with the name of the representative gene. Red nodes – genes whose promoters contain the G-box motif; white nodes – genes whose promoters lack the G-box motif.

The bZIP transcription factor family includes 75 members in Arabidopsis that regulate diverse signaling processes in plants [28]. bZIP TFs predominantly bind to the G-box (CACGTG) motif in promoters. We analyzed how the G-box motif is distributed across the gene co-expression network. Out of the 10,385 genes analyzed, 497 exhibited a pValue for the G-box lower than 0.001 (Table S2), while only 5 genes on average were recovered in permutation experiments with randomized promoter sequences. The estimated false discovery rate (FDR) is 1%. A sub-network for these 497 genes is extracted from the original gene co-expression network (Figure 2). Out of the 497 genes in the sub-network only 291 harbor the G-box motif. The remaining 206 genes are represented in the sub-network because their neighbors possess the G-box motif. Within the G-box sub-network, several densely connected sub-groups of genes or expression modules were identified. Functions of genes in the sub-network are illustrated by their enriched GO term (Figure 2, Table 1).

**Table 1:** GO enrichment of co-expression modules identified in the G-Box sub-network*.

Module	Enriched GO (or notes)	pValue
I	Phytosynthesis	6.24E-10
II	circadian rhythm	1.62E-06
III	cold acclimation	9.28E-15
IV	flavonoid biosynthetic process	4.56E-07
V	response to abscisic acid stimulus	7.31E-17
VI	seed development	3.37E-19
VII	response to jasmonic acid stimulus	1.23E-08
VIII	response to endoplasmic reticulum stress	3.88E-16
IX	glycosinolate biosynthetic process	1.84E-22
X	response to chitin	2.87E-12
XI	starch matebolic process	2.23E-10
XII	response to heat	6.81E-06
XIII	(seed specific expression)	N/A
XIV	(root specific expression)	N/A

**Figure 3:**
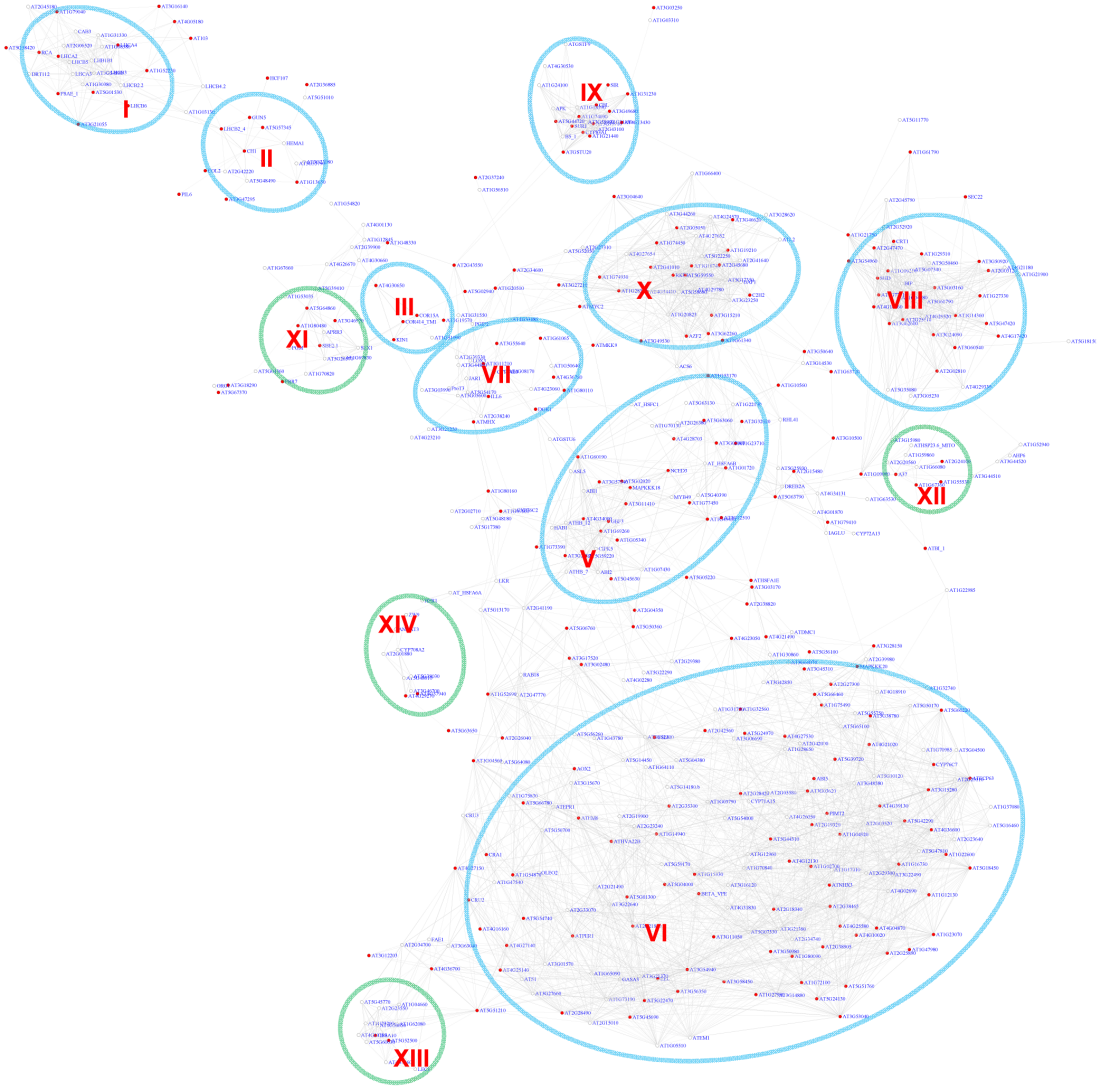
A sub-network for the G-box motif based on the motif position bias analysis. Genes were identified in the motif position analysis with z-score> = 3. Thirteen modules were identified. Among them, 9 modules (circled in blue) were also identified *via* the motif enrichment analysis (See Figure 2), while 4 modules (circled in green) represent additional modules identified *via* the motif position analysis. Red nodes – genes whose promoters contain the G-box motif; white nodes – genes whose promoters lack the G-box motif.

* GO enrichment was calculated according to the modules in Figure 2 (Modules I to X) and Figure 3 (Modules XI–XIV). See Table S3 for gene IDs within the modules.

Our analyses identified 10 gene modules that are regulated by various developmental or environmental cues such as abiotic and biotic stress, pathogen elicitors, hormones, and different light regimes (Figure 2, Figure S1, Table 1, and Table S3). Module V, VI, and VIII included genes that are known to be regulated by bZIPs in ABA response pathways [29], [30], embryogenesis [31]–[33], and the ER stress response [34]–[36].

Interestingly, Module V includes genes that are induced by the bacterial pathogen *Pseudomonas syringae* pv tomato (*Pst*) DC3000 but repressed by the DC3000 hrcc^−^ strain that lacks the type III secretion system used to deliver effector proteins into plant cells. This indicates that the *Pst* DC3000 pathogen appears to deliver effectors that stimulate ABA signaling pathways through bZIP transcription factors as reported before [37]. In contrast, Module VI includes ER stress genes that are also induced by various pathogens and elicitor treatments. Several genes in Module X are previously categorized as common stress responsive genes [38] but TFs that regulate these genes via the G-box motif have yet to be identified. Thus, Module X identified here is a novel module requiring further studies.

Interestingly, some bHLH transcription factor family members also bind to the G-box motif [39]. PIF3 and PIF4 bHLH transcription factors bind to G-box containing photosynthesis genes and the circadian rhythm genes LYH and CCA1, indicating Module I and II's regulation by bHLH factors [40], [41]. Module I genes were induced by long exposure to light and Module II genes were induced by short exposure to light. Another bHLH protein AtMYC2 also binds to the G-box element and regulates genes in the jasmonate signaling pathway [42]–[44] which were enriched in module VII. The genes in module IX were enriched for functions in glucosinolate biosynthesis including SUR1 that might be negatively regulated by AtMYC2 [43], [45].

As an independent measure, we carried out module discovery for the G-box motif *via* motif position bias analysis. 519 out of 10,385 genes analyzed show a z-score for G-box larger than or equal to 3. A sub-network for these genes was extracted (Figure 3). On an average, only 1.3 genes were identified with a z-score> = 3 in permutation experiments with an FDR of 0.3%. Interestingly, this method recovered 9 out of the 10 modules that were also identified *via* the pValue method (Figure 2). Modules derived by either method shared a large number of genes, demonstrating the reliability of the analysis. Four additional modules emerged, among them two potentially novel modules regulated by the G-box motif: Module XII is enriched for heat shock proteins and Module XIV contains genes specifically expressed in roots.

The majority of the modules identified in our analyses for the G-box motif are consistent with previous studies that focused on individual pathways. In addition, we discovered three novel modules. Importantly, many genes were identified here as part of the known modules for the first time (Table S3). In addition, our analysis successfully places these genes in a signaling framework that will facilitate further studies on biological functions. Another notable observation is detection of an overlap of the modules for ABA signaling and jasmonate signaling (Figure 2), suggesting that the regulatory circuits to which these genes respond might be under the control of these two hormones. The interaction and binding of bZIP or bHLH transcription factors with the G-box motif in the promoters of these genes might lead to competition. In fact, antagonistic interaction between the two hormones has been reported before [46], [47].

### Expression modules regulated by the MYB motif CCwACC in a co-expression network

**Figure 4:**
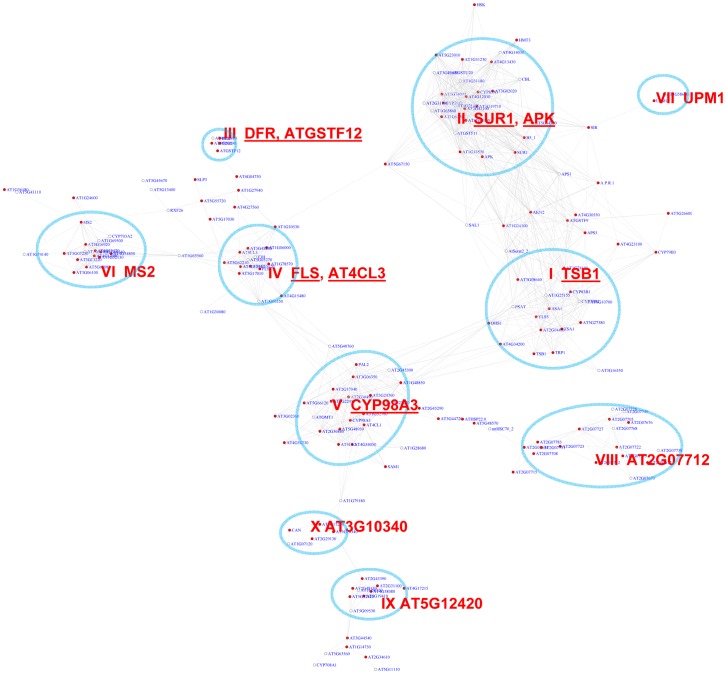
A sub-network for the MYB motifs based on motif enrichment analysis. Genes were identified in the motif enrichment analysis with a pValue for MYB motifs < = 0.01. Ten modules were identified, labeled with the name of the representative gene. Red nodes – genes whose promoters contain the MYB motif; white nodes – genes whose promoters lack the MYB motif.

In *Arabidopsis*, the MYB transcription factor family includes >190 members that regulate diverse functions [48], [49]. We analyzed distribution of two MYB binding motifs, CCwACC and ACCwACC (with “w” standing for “A” or “C”) [50], [51], across the co-expression network. In the network, 243 genes show pValues for CCwACC or ACCwACC lower than 0.01 (Table S2). A sub-network for these genes is shown in Figure 4.

An inspection of the sub-network revealed 10 expression modules (Figure 4, Table 2). A number of these modules are known to function in biosynthesis of various secondary metabolites such as flavonoid (module III), glucosinolate (II), indole derivative (I), anthocyanin (IV), and phenylpropanoids (VIII). Their expression pattern (Figure S2) clearly highlights the activation of diverse metabolic modules in Arabidopsis to cope with distinct environmental stresses. For example, Module V genes were highly induced in response to pathogen elicitors and the bacterial pathogen *Pst* DC3000, possibly representing their function in the basal innate immune response. Genes in this module are implicated in different steps of lignin biosynthesis pathway. Module I genes were up-regulated by broader stimuli including methyl jasmonate, the oomycete pathogen *Phytophthora*, and the fungal pathogen *Botrytis*. In contrast, the glucosinolate genes in Module II were universally repressed by pathogens. Module VII appears to operate in nitrogen metabolism based on the presence of UPM1 and AT3G58610 genes in this module. Together, the functions collected in these modules are consistent with previous reports about MYB-mediated regulation of diverse metabolic pathways [48], [52]–[60]. Three of the modules (VI, IX, & X) in the sub-network are involved in tissue development. Module IX contains genes specifically expressed in roots and seeds (Figures S3), indicating a novel module that might control root and seed development.

**Table 2:** GO enrichment of co-expression modules identified in the MYB motif sub-network*.

Module	Enriched GO (or notes)	pValue
I	indole derivative biosynthetic process	5.49E-21
II	glucosinolate biosynthetic process	1.42E-26
III	flavonoid biosynthetic process	1.17E-17
IV	anthocyanin biosynthetic process	1.33E-06
V	phenylpropanoid metabolic process	1.69E-16
VI	pollen exine formation	1.40E-16
VII	cellular nitrogen compound biosynthetic process	2.86E-04
VIII	respiratory electron transport chain	1.18E-04
IX	(specifically expressed in root and late embryo)	N.A.
X	cell wall polysaccharide biosynthetic process	3.59E-05
XI	abscisic acid mediated signaling pathway	2.46E-04
XII	response to hypoxia	1.92E-05
XIII	sexual reproduction	1.87E-08
XIV	nitrate transport	1.08E-06
XV	response to auxin stimulus	4.80E-14
XVI	response to blue light	7.35E-05
XVII	wax biosynthetic process	8.60E-09
XVIII	(carpel specific expression)	N.A.

**Figure 5:**
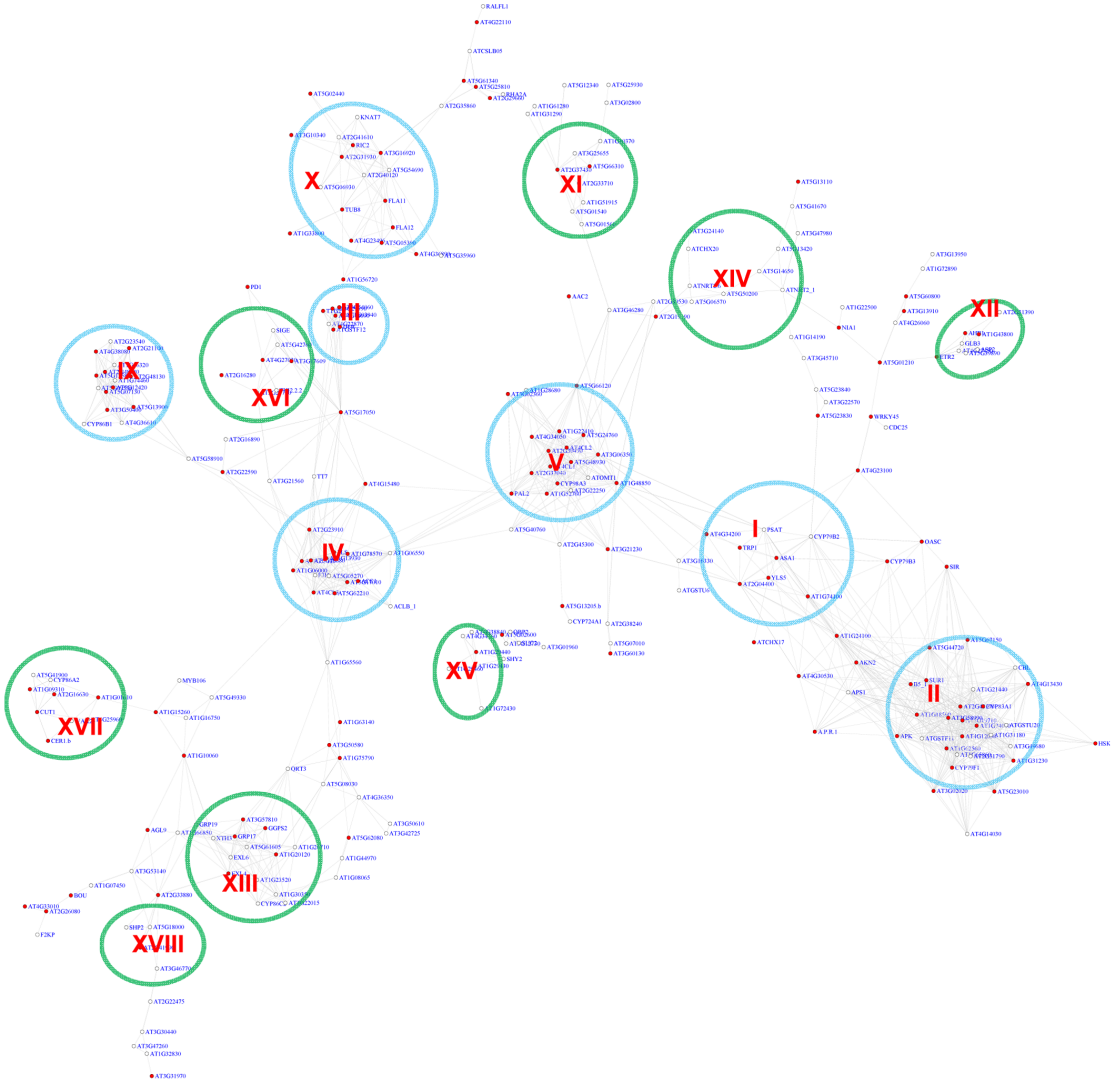
A sub-network for the MYB motifs based on motif position bias analysis. Genes were identified in the motif position analysis with z-score> = 2.2. Sixteen modules were identified. Among them, 7 modules (circled in blue) were also identified *via* the motif enrichment analysis (See Figure 4), while 8 modules (circled in green) represent additional modules identified *via* the motif position analysis. Red nodes – genes whose promoters contain the MYB motif; white nodes – genes whose promoters lack the MYB motif.

* GO enrichment was calculated according to the modules in Figure 4 (Modules I to IX) and Figure 5 (Modules IX–XIV). See Table S3 for gene IDs within the modules.

We also noted that module II of the MYB sub-network shared genes with module IX of the G-box sub-network. For example, SUR1 and CYP83A1 genes (Figure 2 and Figure 4). Gene DFR in module III also appears in module IV of the G-box sub-network. These results indicate some of the genes in these modules are regulated by both the G-box motif and the MYB motif.

The position bias analyses of the MYB motif identified 348 genes in the co-expression network with z-scores for the CCwACC or ACCwACC larger or equal to 2.2. For genes with z-score between 2.2 and 3, it is required that there are at least 5 instances of the motifs within the promoters of that gene and its neighbor genes. A sub-network for these genes is shown in Figure 5. This sub-network revealed 15 modules. Seven of these modules were also identified *via* the pValue method (Figure 4). In the remaining 8 modules 2 function in known MYB-regulated pathways: nitrate transport (XIV) and wax biosynthesis (XVII). Three of the modules are novel and include genes responding to ABA (XI), auxin (XV), and hypoxia (XII).

To assess the FDR in the MYB motif analysis, permutation experiments were conducted with randomized promoter sequences. The permutation was performed 15 times. In each permutation, motif enrichment analysis was conducted for the MYB motif, and the genes with pValue< = 0.01 were used to extract a sub-network from the entire gene co-expression network. A typical sub-network is shown in Figure S4. On average 2.7 gene modules were recovered that each contained at least 6 genes from each permutation. Therefore, the FDR for MYB motif module identification is 2.7 out of 10 or 27% in the motif enrichment analysis. Similarly, in the motif position bias analysis, on average 3.1 gene modules with > = 5 gene numbers were identified among genes with z-score> = 2.2 from each permutation (Figure S5). Thus, there might be up to 3.1 false discovered modules or a FDR of 21% (3.1/15) in the analysis based on position bias. Additionally, in each permutation, only 1 gene on average was recovered with both pValue< = 0.01 and z-score> = 2.2, and no gene modules was identified that fulfills both requirement. This indicates no falsely discovered modules exist among the 7 MYB-related modules recovered by both methods.

### Expression modules regulated by the W-box motif

**Figure 6:**
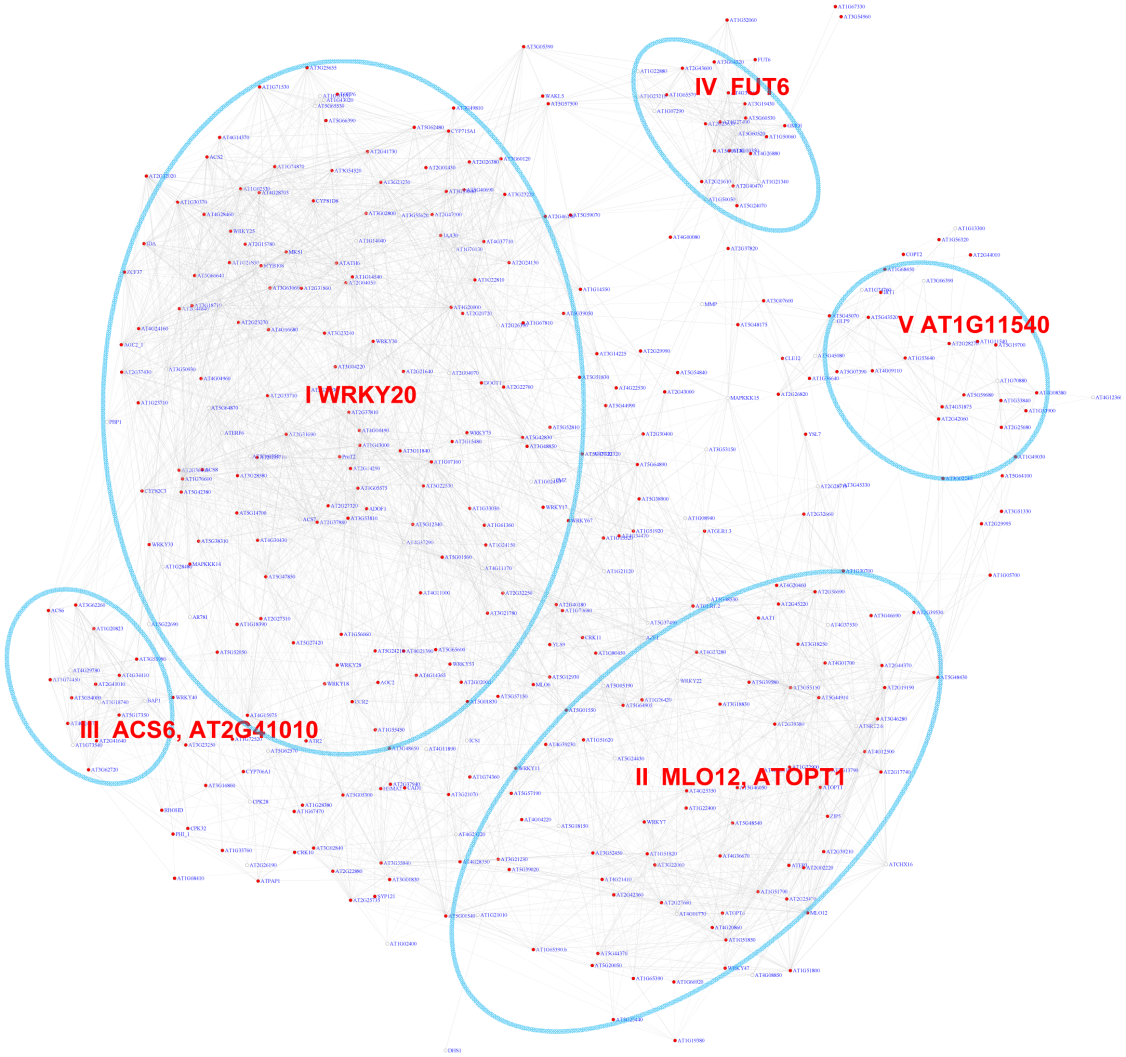
A sub-network for the W-box motif based on the motif enrichment analysis. Genes were identified in the motif enrichment analysis with pValue for the W-box motifs < = 0.001. Five modules were identified and labeled with the name of the representative gene. Red nodes – genes whose promoters contain the W-box motif; white nodes – genes whose promoters lack the W-box motif.

The WRKY transcription factors play important roles in plant defense. They bind to the W-box motif [61]. The core sequence of the W-box motif is TTGACy (with “y” standing for “A” or “G”), but various variant forms of the sequence also show binding affinity to WRKY proteins [62]. Here, we analyzed the W-box motif variant kTTGACy (with “k” standing for “G” or “T”) identified in our previous study [27]. There are 388 genes whose pValues for this W-box motif is less than 0.001 with a FDR of 1.1%. A sub-network for these genes is shown in Figure 6. From this sub-network, five expression modules can be recognized (Table 3). The majority of the genes in modules I and II are regulated by pathogen responses. The genes in Module II are primarily induced by Microbe Associated Molecular Patterns (MAMPs) or by pathogens, while the genes in module I were also strongly induced by salinity stress in Arabidopsis roots (Figure S6). Interestingly, genes in Module II were repressed by *Pst* DC3000 at 6 hour post infection. However, at the same time point, these genes were not repressed by the DC3000 hrcc^−^ mutant (a mutant that's unable to deliver effectors into plant cells) [26]. Thus, it appears that the pathogen *Pst* DC3000 actively delivers effectors into plant cells that interfere with plant signaling pathways and suppress the induction of these genes, presumably for the benefit of the pathogen.

**Table 3:** GO enrichment of co-expression modules identified in the W-box motif sub-network*.

Module	Enriched GO (or notes)	pValue
I	response to chitin	7.66E-14
II	defense response	4.38E-07
III	response to chitin	2.01E-05
IV	cell wall organization or biogenesis	5.18E-04
V	(root specific expression)	N/A
VI	(root specific expression)	N/A
VII	(early slique specific expression)	N/A

**Figure 7:**
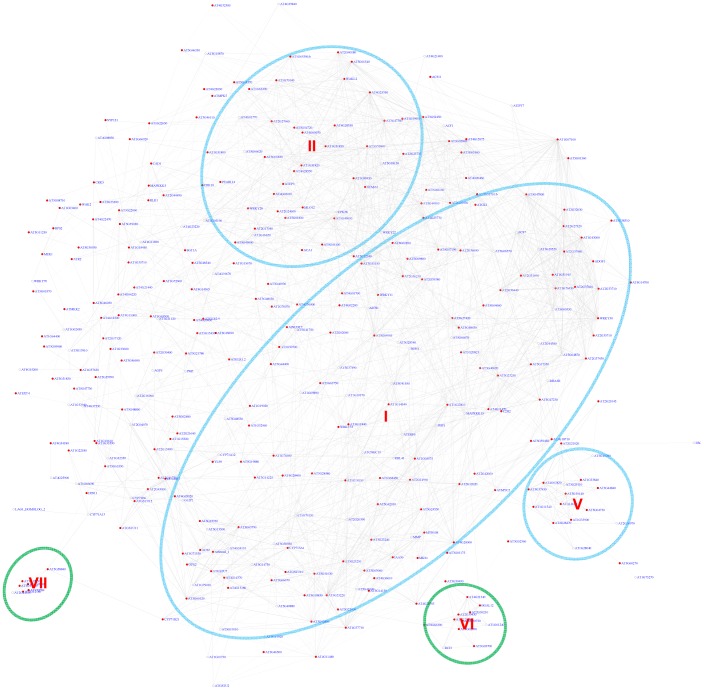
A sub-network for the W-box motif based on motif position bias analysis. Genes were identified in the motif position analysis with z-score> = 3. Five modules were identified. Among them, 3 modules (circled in blue) were also identified *via* the motif enrichment analysis (See Figure 6), while 2 modules (circled in green) were additional modules identified *via* motif position analysis. Red nodes – genes whose promoters contain the W-box motif; white nodes – genes whose promoters lack the W-box motif.

* GO enrichment was calculated according to the modules in Figure 6 (Modules I to V) and Figure 7 (Modules VI–VII). See Table S3 for gene IDs within the modules.

The majority of genes in module III can be characterized as common stress responsive genes because they are induced by different types of abiotic or biotic stress [38]. Interestingly, the majority of genes in module IV are specifically expressed in the roots under normal growth condition (Figure S7) but are repressed by salinity or osmotic stress in roots (Figure S6). In contrast, these genes do not respond to MAMPs or pathogen treatments. These observations raise the possibility that WRKY-mediated signaling might regulate root development. Consistent with these observations, WRKY75 has a function in root hair development [63]. However, any regulatory influence of WRKY75 on genes in module IV has not yet been analyzed. Finally, module V genes are also specifically expressed in roots (Figure S7) and no specific function for WRKY in the regulation these genes are known.

Using the motif position bias analysis, 357 genes were identified with a z-score> = 3 and with a FDR of 2.4%. The recovered modules included 3 modules (I, II, V) identified by the motif enrichment method and 2 additional modules with genes specifically expressed in roots (VI) or siliques (VII) (Figure 7 and Figure S7).

### Expression modules regulated by the Site II element motif TGGGCy

**Figure 8:**
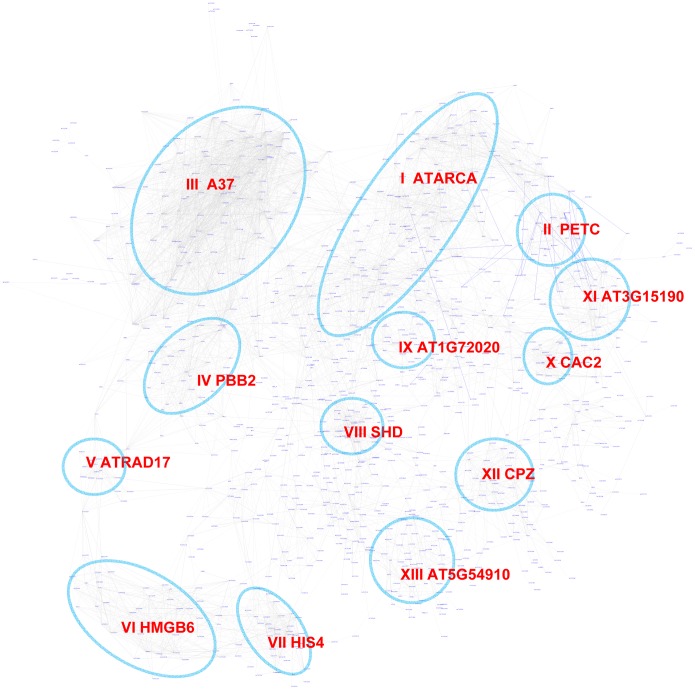
A sub-network for the site II element motif based on motif position bias analysis. Genes were identified in the motif position analysis with z-score> = 3. Thirteen modules were identified, labeled with the name of the representative gene. Red nodes – genes whose promoters contain the site II element motif; white nodes – genes whose promoters lack the site II element motif.

The Site II element motif TGGGCy, bound by TCP transcription factors is present in the promoters of many cell-cycle genes, ribosomal protein genes, and nuclear-encoded mitochondrial protein genes [64]–[67]. Our motif position bias analysis resulted in 1,161 genes with z-scores for TGGGCy larger or equal to 3 with a FDR of 0.4%. The sub-network for these genes is shown in Figure 8. Thirteen modules were identified from the sub-network (Table 4). Consistent with previous reports [64]–[67], modules enriched with cell-cycle genes (V, VI, VII), ribosomal proteins genes (I), and mitochondrial proteins genes (IX) were identified. Our analysis revealed that some nuclear-encoded chloroplast genes may also be regulated by the site II element motif (module II, XI, XII). Additionally, two novel modules (III, VIII) harbor genes functioning in protein folding and one (IV) contains genes encoding members of the proteasome complex. Yet another novel module (X) includes a number of fatty acid biosynthetic genes. Thus, our analysis indicates that site II element motif might regulate a broader array of biological processes than previously thought. Many of the functions that are highlighted show strong relationships to housekeeping functions of plant cells.

**Table 4:** GO enrichment of co-expression modules identified in the W-box motif sub-network*.

Module	Enriched GO	pValue
I	cytosolic ribosome	2.23E-52
II	plastid part	2.81E-21
III	response to heat	3.62E-37
IV	proteasome complex	7.37E-66
V	DNA repair	9.05E-05
VI	DNA replication	9.44E-31
VII	chromatin assembly	3.24E-46
VIII	unfolded protein binding	6.20E-12
IX	respiratory chain	3.08E-12
X	fatty acid biosynthetic process	1.48E-14
XI	chloroplast part	2.38E-21
XII	Chloroplast	1.32E-08
XIII	Nucleolus	2.44E-10

* GO enrichment was calculated according to the modules in Figure 8. See Table S3 for gene IDs within the modules.

Using the motif enrichment analysis method, 161 genes were recovered with pValue< = 0.001 for the motif TGGGCy at a FDR of 3.6%. Therefore, for the site II element motif, the position bias analysis recovered more genes and performed better than the motif enrichment analysis.

### A combined sub-network incorporating gene expression modules regulated by the G-box, MYB, W-box, or the site II element motif

**Figure 9:**
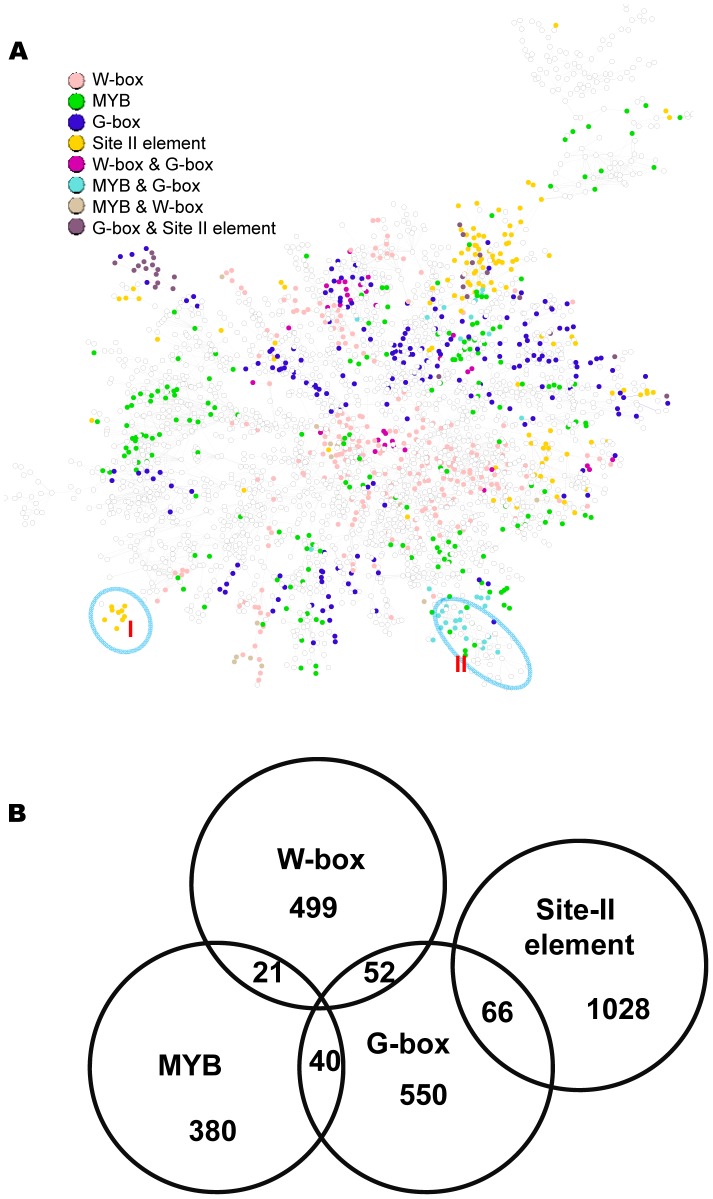
A sub-network with the top 6,000 co-expressed gene pairs extracted from the whole gene co-expression network. (A) Genes identified to be regulated by the G-box, MYB, W-box, and site II element motifs are spread across this sub-network, as indicated by the colors of the nodes. Nodes without colors are genes not identified to be regulated by these motifs. Circled are two modules that recapitulate the results from single motif analysis. (B) A Venn diagram showing the number of genes regulated by individual motifs or by combination of two motifs.

The above analysis identified gene expression modules for individual motifs. Here, these modules were incorporated into a single network. Shown in Figure 9A is a sub-network consisting of the top 6,000 co-expressed gene pairs from the original GGM network (the whole GGM network is too big to depict here). Among the 3,756 genes in this sub-network, 1,056 (28%) are regulated by at least one of the four motifs. Gene modules regulated by the W-box motif appear in multiple clusters across the network. The modules regulated by G-box, MYB, or site II elements have similar distribution pattern. A number of modules within the network are regulated by two motifs: MYB & G-box, W-box & G-Box, or G-box & site II elements (Figure 9B). These modules are similar to those identified via single motif analysis. For example, module I from this analysis is regulated by the site II element and shares many genes with the site II element module VI from the single motif analyses (see Figure 8). Module II is regulated by both the G-box and MYB motifs and shares many genes with the G-box module IX (see Figure 2) and the MYB module II (see Figure 4) from single motif analyses. The structure of the combined motif sub-network is more complex than the one derived from single motif analyses, while the single motif analyses provide the basis to reveal the modular structures within this network.

### Comparison between the bottom-up and top-down approaches for module discovery

To compare our bottom-up module discovery approach described here with the top-down approach, we used previously published Arabidopsis Gene Co-expression Network (AGCN) generated from 1,094 Affymetrix ATH1 microarray data sets via the AtGenExpress project [9]. The AGCN network contained 6,206 genes and was clustered into 527 modules using the MCL algorithm via a top-down approach [9]. Using the same motif enrichment and motif positions bias analysis employed in our bottom-up approach, we identified AGCN modules that are regulated by the G-box, MYB, WRKY, and the site II element motifs (Table S4). The results of comparative analyses are shown in Table 5 and Figures S8, S9, S10, S11, S12, S13, S14, S15. The two approaches were considered to share a common identified motif-driven module if the respective modules from each approach share common genes between them. 7 out of the 14 modules regulated by G-box motif identified via our bottom-up approach were not recovered by in the AGCN network using the top-down approach (Figure S8 and S9). These include the modules responding to ABA (V) and heat shock (XII), and modules related to flavonoid (IV) and glucosinolate (IX) metabolism. Similarly, our bottom-up method also identified 10 unique modules for the MYB motif (Figure S10 & S11). While both methods identified similar number of modules for WRKY motif (Figure S12 & S13), the top-down method recovered 2 more distinctive modules for the site II elements (Figure S14). Overall, more unique modules were identified via our bottom-up approach.

**Table 5:** Comparison between the bottom-up approach (for GGM network) with the top-down approach (for AGCN network) on module discovery.

Motif	# of modules identified via bottom-up approach for the GGM network	# of modules identified via top-down approach for the AGCN network	GGM modules shared by AGCN*	GGM modules not shared by AGCN*	# of AGCN modules not shared by GGM**
**G-Box**	14	5	7 - I, II, VI, VII, VIII, X, XI	7 - III, IV, V, IX, XII, XIII, XIV	0
**MYB**	18	11	8 - I, II, IV, VIII, X, XII, XIV, XV	10 - III, V, VI, VII, IX, XI, XIII, XVI, XVII, XVIII	3
**WRKY**	7	8	6 - I, II, III, IV, V, VI	1 - VII	1
**Site II element**	13	21	11 - I, II, III, VI, VII, VIII, IX, X, XI, XII, XIII	2 - IV, V	4

* Shown are the total number of modules and their id according to those in Figure 3–8. See Figure S8, S9, S10, S11, S12, S13, S14 for more details.

** See Table S4 for more details.

The MCL clustering program used in the top-down approach on AGCN network generated 3 large clusters (cluster No. 1, 2, and 3) with more than 500 genes in each cluster (Table S4) [9]. These three clusters include 2,684 genes that represent 43% of all the genes in the AGCN network. These clusters are large and include a mix of real targets of modular regulation with many non-targets. Therefore, prioritizing true target genes from this large cluster size for downstream analyses is not straightforward. For example, the largest cluster (No. 1) of the AGCN network contains 1,362 genes. The enrichment of the G-box motif in this cluster suggests that all genes within the cluster are regulated by this motif. In contrast, our bottom-up approach analysis on the GGM network revealed that only 62 genes out of these 1,362 genes are regulated by the G-box motifs (Figure S15). These G-box regulated genes did not spread evenly across the whole sub-network, but occupied certain distinctive sub-domains within it. Thus, our bottom-up approach was able to differentiate the genes potentially regulated by G-box motif from those non-targets, resulting in a more refined and precise gene regulation model than those obtained via the top-down approach.

### A rapid screening system to validate transcription factor-promoter interaction *in vivo*

From our analysis it is apparent that a single motif can regulate multiple expression modules. These modules might be regulated by different transcription factors (TFs) from the TF family which bind to that motif. For example, the 19 modules identified for the MYB motif (Figures 4 and 5) can be driven by different MYB transcription factors. An important task that remains for our understanding of transcriptional networks will be to distinguish the specificities within a TF family, i.e. which member or members of TF family drive the expression of individual modules. At the same time, the TFs that regulate genes in the module might not be a part of the modules themselves in the co-expression network. This is because TFs themselves may not be regulated at the transcriptional level but may be regulated at the translational or protein turnover levels and thus might have expression patterns different from the genes in the modules. Therefore, analyzing a co-expression network in isolation is not sufficient to identify the TFs responsible for regulating the expression modules.

**Figure 10:**
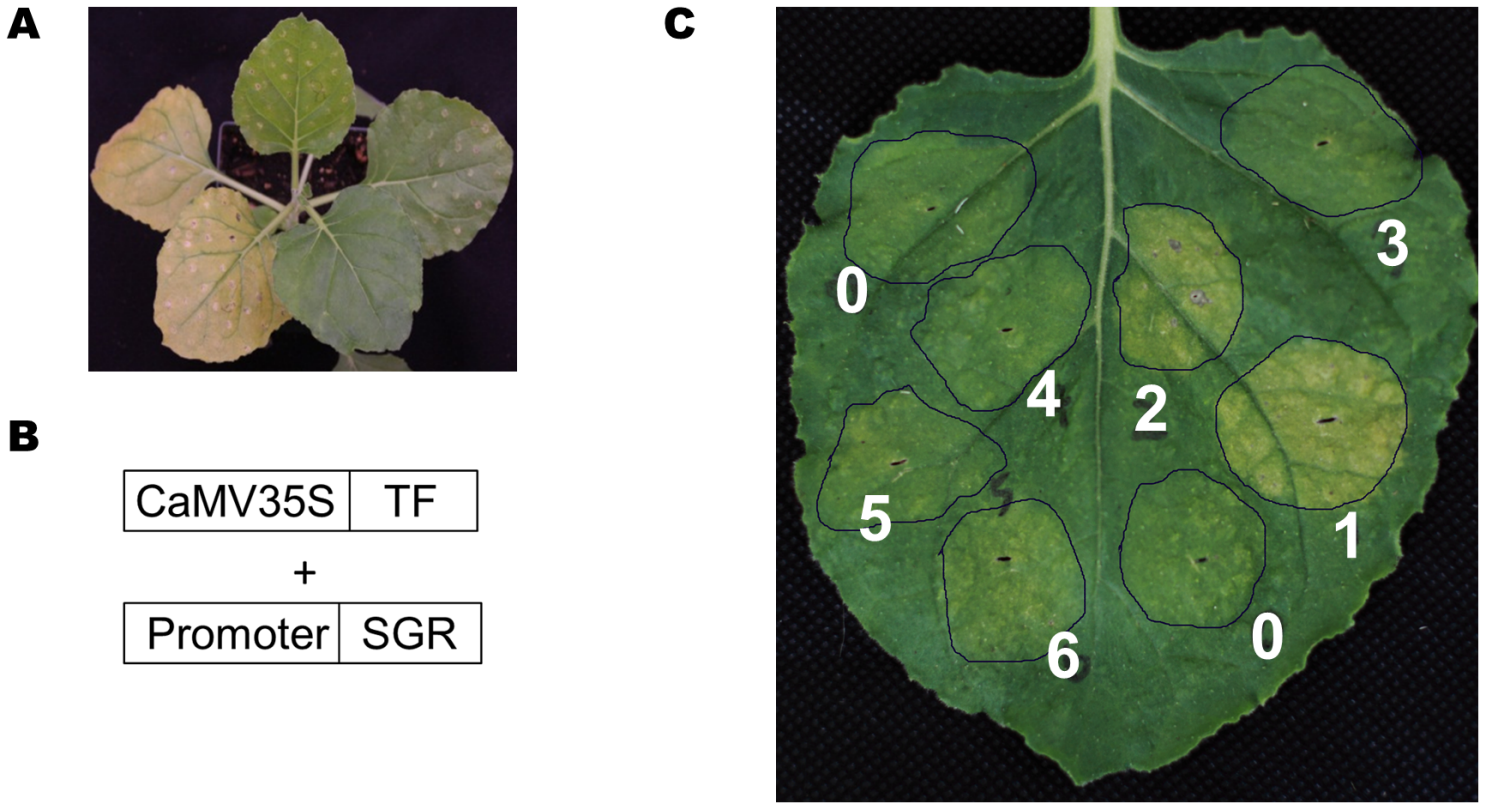
A TF-promoter screening system based on the SGR gene. (A) Transient overexpression of the SGR gene (left two leaves) in *N. benthamiana* induced yellowing, while a control gene did not (two leaves, upper right). (B) The design scheme for the screening system. (C) Transient over-expression of the SUR_Promoter::SGR construct together with 6 different MYBs (#1 to #6), and an actin gene as negative control (#0). Only MYB28 (#1), MYB29 (#2) induced yellowing.

To this end, we developed a rapid screening system to test the transcription factor–promoter interactions. The setup employs the Arabidopsis At4g22920 gene that encodes stay green (SGR) protein as a reporter. SGR protein is required for dismantling chlorophyll-protein complexes, leading to chlorophyll degradation [68], [69]. Transient over-expression of the *SGR* gene under the control of CaMV 35S promoter induces yellowing of leaves in *Nicotiana benthamiana* (Figure 10A).

In the screening system, the *SGR* gene was placed behind a promoter of interest and transiently co-expressed with a selected TF in *N. benthamiana* (Figure 10B) (see Materials and Methods for details). If the over-expressed TF can bind to the promoter of interest and drive the expression of *SGR* gene, the infiltrated *N. benthamiana* leaves will turn yellow (Figure 10A). In a pilot experiment, the SUR1 gene promoter was linked to the SGR gene and co-expressed with seven different Arabidopsis MYB TFs or an actin gene as a negative control. Only AtMYB28 and AtMYB29 caused leaf yellowing (Figure 10C; spot #1 and #2). Thus, this straightforward screen established interaction between MYB28 and MYB29 transcription factors and the SUR1 promoter.

Next, we were interested in determining which MYB TFs regulate the five expression modules (Figure 4) involved in different secondary metabolic pathways. Using the SGR screening approach, eight promoters from these 5 different expression modules (Figure 4) were selected, and screened against 82 different Arabidopsis MYB TFs. The TSB1 promoter of Module I displayed exceptionally high basal expression levels in the leaves and was excluded from further experiments. The analyses identified 34 interactions between 18 AtMYB TFs and 7 promoters (Table 6). For each promoter, at least one MYB protein was identified as driving its expression.

**Table 6:** TF-Promoter interaction verified by SGR- and luciferase-based screening.

Expression Module No.		IV	III	II	II	V	IV	III
Promoter Gene Name		FLS	ATGSTF12	SUR1	APK	CYP98A3	AT4CL3	DFR
Promoter Gene AGI		AT5G08640	AT5G17220	AT2G20610	AT2G14750	AT2G40890	AT1G65060	AT5G42800
**TF_AGI**	**TF Name**							
**AT5G60890**	**MYB34**	0	0	++*	++*	0	0	++
**AT3G49690**	**MYB84, RAX3**	0	++*	0	0	0	0	0
**AT1G74430**	**MYB95**	0	0	0	++*	0	0	0
**AT1G18570**	**MYB51, HIG1**	0	++*	++*	++*	0	0	0
**AT5G61420**	**MYB28, HAG1**	0	+*	++*	++*	++*	0	0
**AT5G07690**	**MYB29, PMG2**	+	0	++*	++*	0	0	0
**AT3G05380**	**ALY2**	0	0	0	0	0	0	0
**AT1G48000**	**MYB112**	0	0	0	0	0	0	0
**AT2G16720**	**MYB7**	0	0	0	0	0	0	0
**AT1G66230**	**MYB20**	0	++	0	0	++	0	0
**AT1G34670**	**MYB93**	0	++*	0	0	0	0	0
**AT3G01140**	**MYB106**	0	++	0	0	++*	0	0
**AT5G62470**	**MYB96**	0	+*	0	0	0*	0	0
**AT5G10280**	**MYB92**	0	++*	0	0	+*	0	0
**AT5G16770**	**MYB9**	0	++	0	0	+*	0	0
**AT5G26660**	**MYB86**	0	++	0	0	0	0	0
**AT3G62610**	**MYB11**	++	+*	0	0	++	++	0
**AT5G07700**	**MYB76**	0	0	0	0	0	0	0
**AT3G16350**		0	0	0	0	0	0	++
**AT2G31180**	**MYB14**	0	0	0	0	++*	0	0
**AT3G23250**	**MYB15**	0	0	0	0	++*	0	0
**AT4G34990**	**MYB32**	0	0	0	0	++*	0	0

++ - strong interactions identified in the SGR assay.

+ - weak interactions identified in the SGR assay.

* indicates interactions confirmed by luciferase-based assay.

As a further validation of our SGR reporter assay, a luciferase-based assay was performed to measure the promoter activity [70], [71]. Four selected promoters were cloned in front of the luciferase gene and co-expressed with different AtMYB TFs in *N. benthamiana*. Luciferase activities were then measured 48 or 72 hours later as an indication of the promoter activities (Figure S16). We tested 29 of the 34 interactions identified using the SGR system, and confirmed 23 of them. This demonstrates the usefulness of the rapid SGR-based screening system and its value in the analysis and verification of predictions made by the program.

Among the interactions recovered by both reporter systems are the interaction between the SUR1 and APK promoters and several regulators of glucosinolate synthesis, including ATR1, HIG1, HAG2, and PMG2, which is consistent with previous reports [53], [54]. The gene *CYP98A3* is from module V of the MYB sub-network. Module V is enriched with lignin biosynthesis genes that are induced by pathogen treatment. This is consistent with previous reports that infection by pathogens induced lignification in plants [72]–[74], although mechanistic details are not known. MYBs are important regulators of lignin biosynthesis [75] but the exact MYB(s) that regulate pathogen induced lignification have yet to be identified. Our results showed that several MYBs drive the expression of the CYP98A3 promoter (Table 6, Figure S16). Among them, the *MYB14*, *MYB15* and *MYB32* genes themselves were also induced by pathogen treatments (Figure S17). These MYBs might act as master regulators of the lignification process in the response leading to pathogen resistance.

## Discussion

We describe a bottom-up strategy to identify gene expression modules from gene co-expression networks that are regulated by known promoter motifs. Two independent methods were used to identify genes belonging to modules regulated by specific motifs: based on motif enrichment and motif position bias. For the G-Box, W-Box, and the site II elements, the cut-offs were set at a pValue of 0.001 for motif enrichment analysis and a z-score of 3 for position bias analysis. Many known and a number of novel modules were identified with a FDR of ∼1%, indicating very high confidence. To recover additional modules for the MYB motif, the cut-offs were lowered to 0.01 for the pValue and 2.2 for the z-score. From this, 18 modules were identified with a FDR of 21%–27% representing moderate confidence. However, the overlap of modules between the motif enrichment analysis and the motif position bias analysis for MYBs revealed high confidence. Thus, two different stringency levels may be chosen depending on the nature of the motifs. Even at high stringency levels, our analysis identified more modules than other module analysis based on gene co-expression networks, such as the AGCN network (Table 5) or by Vandepoele et al. [76]. For the G-Box motif, the analysis by Vandepoele et al. [76] recovered modules enriched with GO terms for response to cold, photosynthesis, starch metabolism, and response to ABA. Our analysis identified 14 modules and includes many additional GO-terms (Table 1). The site II element motif analysis by Vandepoele et al. recovered modules enriched for the GO term ribosome biogenesis and assembly. Our analyses identified 13 modules consisting of 6 known and 7 novel modules.

Promoter motifs have long been shown to have position bias towards TSS [27], [77]–[79]. This feature has been widely used as supporting evidence for the validity of a *bona-fide* motif in motif discovery algorithms. For example, the AMADEUS platform calculates localization bias based on a binned enrichment score [80], while the FIRE program uses mutual information to detect motif position bias [79]. Here, a z-score based on uniform distribution was used to measure motif position bias [27]. Our analysis provides evidence that motif position bias could be used as an effective tool to identify gene expression modules. In the four motifs studied here, our analysis based on motif position bias performed as well as (for G-box and W-box motif) or even better (for MYB and the site II element motifs) than analyses based on motif enrichment. For some of the identified motif–module combinations, the motif was localized with position bias within the modules without enrichment. Therefore, application of motif position analysis to other known plant promoter motifs has the potential to lead to the discovery of additional novel signaling modules that so far have escaped recognition.

**Figure 11:**
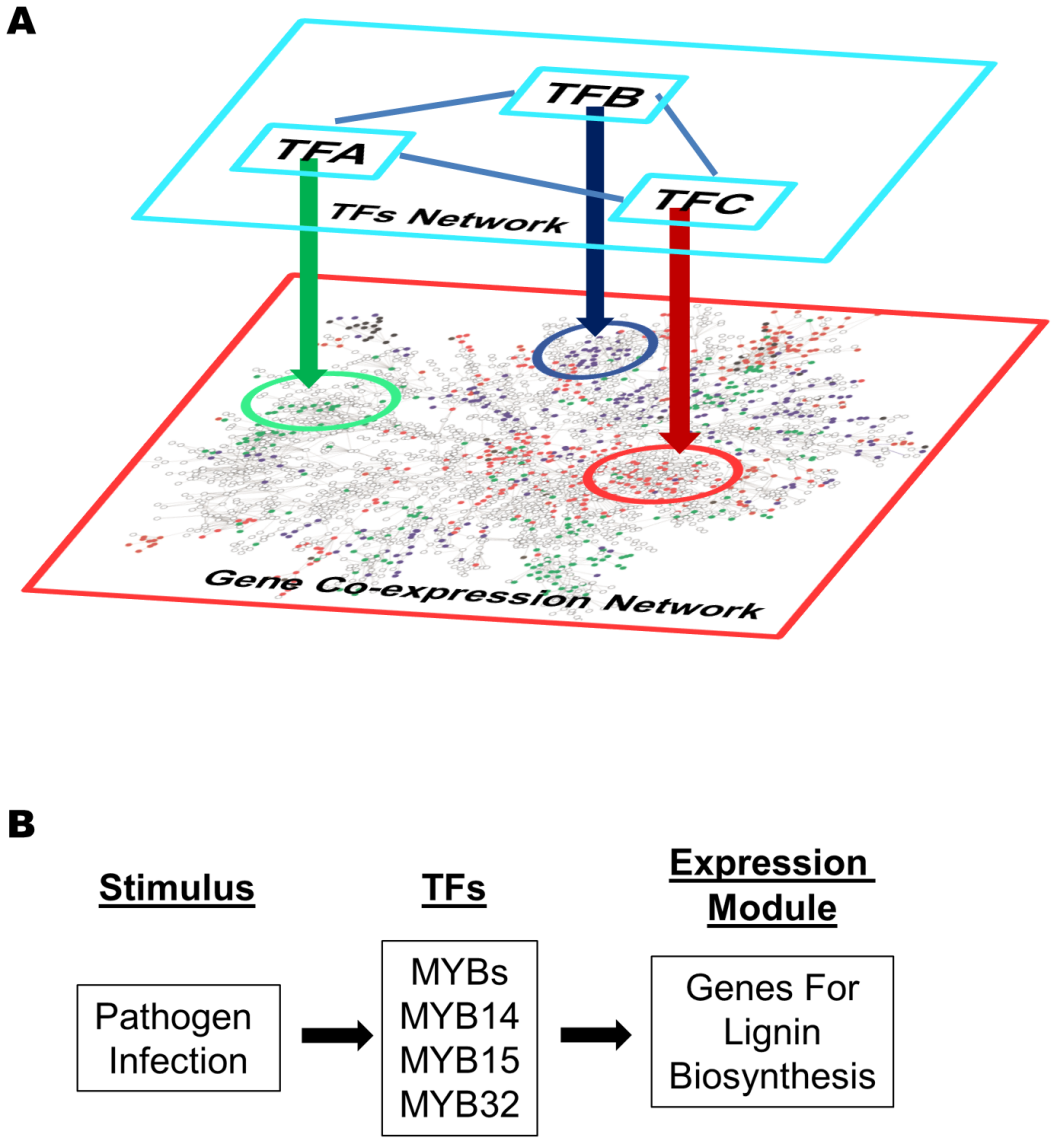
Gene expression modules regulated by transcription factors in a gene co-expression network. (A) Different modules in a gene co-expression network are regulated by different transcription factors (TF-A, TF-B, TF-C etc). These transcription factors are not necessary part of the co-expression network, and they might interact with each other and form a regulatory network of themselves. (B) A gene expression model derived from the network analysis. The MYB transcription factors (MYB14, 15, and/or 32) are activated upon pathogens infection and turn on the expression of down-stream lignin biosynthesis genes (described in Figure 4 Module V).

Our approach to identify motif based gene expression modules presents a novel step to understand the regulatory mechanisms underlying gene co-expression networks. An important task for gene network analysis is to identify hub genes which serve as the key regulators that determine the expression of other genes within the network. Genes with the most number of connections are usually treated as hubs. Here, we argue that for co-expression modules driven by a specific motif, hub genes should be the TFs that bind to the motif and regulate gene expression. These TFs might not be part of the gene co-expression network and can form regulatory networks themselves (Figure 11A). For the modules identified from our analysis, the potential regulatory motif and TF family that govern the structure of a co-expression module can be identified. In turn, the rapid TF-promoter interaction screening system based on the SGR gene provides a fast method to identify the exact transcription factor(s) that drives the expression of a specific module, thus revealing the specificities for the TFs within the same family. For example, our results indicated that the MYB-motif containing SUR1 promoter is only activated by a subset of MYB TFs, while CYP98A3 promoters are activated by another subset of MYB TFs. On the other hand, some MYB TFs do not activate any of the selected promoters whose targets might reside in the MYB modules we have not tested. It is intriguing how such specificities between different MYB TFs and different MYB motif containing promoters are achieved. The specificities might be determined by different MYB motif variants, or the nucleotides flanking the core MYB motifs, or the combinatorial effects from other motifs in the same promoters. As another advantage, our analysis also benefits from an existing library collection generated in our laboratory for the expression of plant proteins in *N. benthamiana* for protein microarray productions including 1,100 Arabidopsis transcription factors [5], [8](Ma et al., unpublished data).

Finally, coupling the gene co-expression network, module analysis, and gene expression visualization provides a powerful way to study gene signaling systems. First, applying gene expression visualization on co-expression modules can easily determine if the response of the genes are mirrored by the same stimulus, i.e. W-box module I, II, III (Figure S6), or whether genes share similar or identical expression pattern in particular tissues, i.e. W-box module IV, V (Figure S7). Second, by comparing different expression modules, general frameworks of signaling pathways can be outlined. For example, Figure S18 shows the expression of three modules induced by pathogens, namely *via* the G-box, MYB, and W-Box motif, respectively. Both MYB and W-Box modules are induced by MAMPs and pathogens, and were repressed by *Pst* DC3000. However, only the W-Box module was repressed by ABA treatment. Therefore, these two modules represent two different branches of the basal immunity pathways regulated by MYB and WRKY transcription factors respectively. The MYB module mainly contains lignin biosynthesis genes and our rapid SGR screening system identified MYB 14, 15, or 32 could be their regulators. A model for such regulation is depicted in Figure 11B which can be further tested using different MYB mutant lines. As discussed before, the bZIP module might be induced by *Pst* DC3000 effector proteins delivered into plant cells *via* ABA pathway. It will be interesting to test the potential repression of the W-Box modules by pathogen effectors in dependence on ABA.

In conclusion, we provide a robust approach useful for the identification of gene co-expression modules regulated by known promoter motifs that can be extracted from gene co-expression networks. These predicted TF-promoter interactions could be verified easily using a novel rapid screening system based on SGR reporter gene expression. The algorithm will be available freely for downloading to aid in the identification of expression modules based on motifs selected by the user.

## Materials and Methods

### Gene network, promoter sequences, and promoter motifs

We used an Arabidopsis gene co-expression network based on the Graphical Gaussian model described before [15], [26]. The software package GeneNet was used when constructing the network [16], [81]. From this network, 120,276 gene pairs with absolute values of partial correlation co-efficient > = 0.05 (pValue< = 7.03E-49) were chosen for the analysis, which contained 16,456 genes (Additional data file 1).

The Arabidopsis promoter dataset was downloaded from TAIR (ftp://ftp.arabidopsis.org/Sequences/blast_datasets/TAIR10_blastsets/upstream_sequences/TAIR10_upstream_1000_20101104). The promoters are defined as the first 1,000 bp upstream of the 5′ UTR or upstream of translation start codon if no 5′ UTR data were available of the 33,602 TAIR 10 gene loci.

Our algorithm works with any promoter motifs described as IUPAC consensus word sequences, consisting of the nuclides A, C, G, T, and wobble nucleotides r (A or G), y (C or T), s(G or C), w (A or T), m(A or C), k (G or T), or n (any base). Many plant promoter motifs are registered as such consensus word sequences in the AGRIS and PLACE databases [82], [83]. We chose four well-known motifs for the current study.

### Motif enrichment analysis

Motif enrichment was assessed based on hypergeometric distribution. For a given motif, a pValue of motif enrichment was calculated for every gene in the network. Suppose a gene and all the genes immediately connected with it form a group of genes with *M* promoters in total, and a motif presents in *m* promoters among them. Within the *K* promoters in the whole Arabidopsis genome, the motif presents in *k* promoters. A pValue for that motif and gene combination is calculated as:
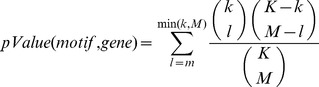



### Motif position bias analysis

Motif position bias towards TSS was assessed based on the uniform distribution [27]. For a given motif, a z-score of motif position bias was calculated for every gene in the network. Suppose a motif appears *n* times in the promoters of a gene and all the immediately connected genes. The locations of these *n* motif instances relative to TSS is *p_1_*,*p_2_*,…,*p_n_*, and their mean value is *p*. A z-score for that motif and gene combination is calculated as:
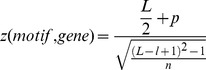
where *L* is the length of the promoters, and *l* is the length of the motif. The motif position is the midpoint of the motif relative to TSS. For orientation, we describe p = 0 as the position at TSS, and p = −1000 at position of 1000 bp upstream of TSS.

### Network visualization and GO analysis

For a given motif, genes with pValue of motif enrichment smaller or equal to cut-off were selected. A sub-network was extracted from the gene co-expression network for these genes. A sub-network can also be extracted for all the genes with z-score value larger or equal to a selected cut-off value. Network visualization was carried out using the neato program with the “stress Majorization” algorithm which is included in the software package Graphviz 2.21 [84], [85]. The lay-out of the sub-network is then visually inspected for modules. GO enrichment analysis was then conducted by genes within these modules.

### Permutation calculations

Permutation experiment on randomized promoters was carried out to measure false discovery rate. Two steps were employed to randomize promoter sequences. First, each of the 33,602 promoter sequences in the TAIR *Arabidopsis* promoter dataset was randomized within itself. The order of nucleotides was completely shuffled but the total numbers of each type of nucleotide were kept the same. Then the resulting promoter sequences were randomly assigned to each of the 33,602 genes without replacement. Gene expression module discovery was then carried out on these randomized promoters and false discovery rate calculated.

We used an in-house developed software package called MotifNetwork to conduct the above mentioned motif enrichment analysis, motif position bias analysis, sub-network extraction, and permutation analysis. The algorithm is provided through our website (http://dinesh-kumarlab.genomecenter.ucdavis.edu/downloads.html) and upon request for academic use.

### Gene expression data

Transcription profiling of *Arabidopsis* gene expression in different tissues or gene expression regulation upon treatments with different abiotic stresses, hormones, pathogen elicitors, pathogens, and different light regimens were obtained from the AtGenExpression project [86], [87]. The data were downloaded from WeigelWorld (http://www.weigelworld.org/resources/microarray/AtGenExpress) and TAIR (http://www.arabidopsis.org/portals/expression/microarray/ATGenExpress.jsp). Data were processed as previously described [38]. Table S5 lists the treatments used in the gene regulation profiling experiment in Figure S1, S2, S6, S17, and S18. Table S6 lists the tissues used in the tissues expression profiling experiments in Figure S3 and S7.

### Transcription factor-promoter interaction analysis

A TF-promoter interaction screen system was developed based on the *stay green* gene (*SGR*). A gateway vector, SPDK2388, was generated with a gateway cassette placed in front of *SGR*. The promoter::*SGR* construct was generated *via* gateway cloning of the selected promoters (1000 bp). Previously, we built an expression library for expressing *Arabidopsis* proteins in plants [5], [8] (Ma et al., unpublished data), which include the 82 TF genes used in this analyses.

For SGR-based screening, selected promoters were cloned into SPDK2388, and transferred into *Agrobacterium tumefaciens* GV2260. Over-night cultures of *Agrobacterium* with selected promoter vectors were centrifuged and re-suspended to O.D_600_ = 0.1–0.3 with infiltration medium (10 mM MgCl_2_, 10 mM MES, 200 mM acetosyringone), and mixed with TFs Agrobacterium O.D_600_ = 1.0. The mixed Agrobacterium cultures were then spot-infiltrated into 5 week-old *N. benthamiana* leaves. The infiltrated spots were inspected at 48 to 96 hours after infiltration for signs of yellowing.

TF-promoter interactions were also analyzed with the dual luciferase system according to the protocol described in [71]. Briefly, selected promoters were cloned into the pBGWL7 [88] vector to make Promoter::LUC cassette, and transferred into *A. tumefaciens* GV2260. The transferred Agrobacteria were then co-infiltrated into 5 week old *N. benthamiana* leaves with Agrobacteria containing a vector to constitutively express *hRenilla* genes and Agrobacteria containing different TFs. Leaf disc of 1 cm in diameter from the infiltrated spot were collected and used for luciferase and Renilla fluorescence measurement using the Dual-Luciferase Reporter Assay System (Promega, Fitchburg, WI) as described in [71].

## Supporting Information

Figure S1 Expression pattern for genes in G-box modules after different treatments. Data according to AtGenExpress.
(PDF)

Figure S2 Expression pattern for genes in five MYB modules after different treatments. Data according to AtGenExpress.
(PDF)

Figure S3 Expression pattern for the genes in three MYB modules in different tissues. Data are represented as relative expression levels. Data according to AtGenExpress.
(PDF)

Figure S4 A typical sub-network for the genes recovered for MYB with pValue< = 0.01 in a permutation expression. Three modules with > = 5 genes were identified. The solid and grey lines indicate gene pairs with top 20% or bottom 20% partial correlation values respectively.
(PDF)

Figure S5 A typical sub-network for the genes recovered for MYB with z-score> = 2.2 in a permutation expression. Five modules with > = 5 genes were identified. The solid and grey lines indicate gene pairs with high or low partial correlation values respectively.
(PDF)

Figure S6 Expression patterns for the genes in five W-box modules upon different treatments. Data according to AtGenExpress.
(PDF)

Figure S7 Expression patterns for the genes in two W-box modules in different tissues. Data are represented as relative expression levels. Data according to AtGenExpress.
(PDF)

Figure S8 Comparison between the GGM network (bottom-up approach) and the AGCN network (top-down approach). The sub-network identified for the G-box motif via motif enrichment analysis for the GGM network shown in Figure 2 is intersected with the G-box modules identified in the AGCN network. Red nodes - genes identified in both methods; white nodes – genes identified only in the GGM network. Circled in grey are modules identified only via the GGM methods. Modules identified in both methods are circled in blue.
(PDF)

Figure S9 The sub-network identified for the G-box motif via position bias analysis for the GGM network shown in Figure 3 is intersected with the G-box modules identified in the AGCN network. Red nodes - genes identified in both methods; white nodes – genes identified only in the GGM network. Circled in grey are modules identified only via the GGM methods. Modules identified in both methods are circled in blue.
(PDF)

Figure S10 The sub-network identified for the MYB motif via motif enrichment analysis for the GGM network shown in Figure 4 is intersected with the MYB modules identified in the AGCN network. Red nodes - genes identified in both methods; white nodes – genes identified only in the GGM network. Circled in grey are modules identified only via the GGM methods. Modules identified in both methods are circled in blue.
(PDF)

Figure S11 The sub-network identified for the MYB motif via position bias analysis for the GGM network shown in Figure 5 is intersected with the MYB modules identified in the AGCN network. Red nodes - genes identified in both methods; white nodes genes identified only in the GGM network. Circled in grey are modules identified only via the GGM methods. Modules identified in both methods are circled in blue.
(PDF)

Figure S12 The sub-network identified for the W-box motif via motif enrichment analysis for the GGM network shown in Figure 6 is intersected with the W-box modules identified in the AGCN network. Red nodes - genes identified in both methods; white nodes – genes identified only in the GGM network. Modules identified in both methods are circled in blue.
(PDF)

Figure S13 The sub-network identified for the W-box motif via position bias analysis for the GGM network shown in Figure 7 is intersected with the MYB modules identified in the AGCN network. Red nodes - genes identified in both methods; white nodes – genes identified only in the GGM network. Circled in grey are modules identified only via the GGM methods. Modules identified in both methods are circled in blue.
(PDF)

Figure S14 The sub-network identified for the site II element motif via position bias analysis for the GGM network shown in Figure 8 is intersected with the site II element modules identified in the AGCN network. Red nodes - genes identified in both methods; white nodes – genes identified only in the GGM network. Circled in grey are modules identified only via the GGM methods. Modules identified in both methods are circled in blue. Blue lines connecting two genes indicated that they have negative correlated expression pattern.
(PDF)

Figure S15 A sub-network extracted for the 1,362 genes in the AGCN cluster No. 1 from the GGM network. Labeled in red are those genes deemed to be regulated by the G-box motif via our bottom-up approach analysis on the GGM network.
(PDF)

Figure S16 The interactions between MYBs and selected promoters. Assays conducted with the dual luciferase system. Plotted are the relative luciferase activities for different MYB plus Promoter::LUC combination. The red lines in each panel indicate the threshold level for interaction. Name on the top of the graph indicates promoter of the gene used in the assay. Different MYBs used are shown below each bar on the X-axis.
(PDF)

Figure S17 Expression patterns for different MYB TFs under different treatments. Data according to AtGenExpress.
(PDF)

Figure S18 Expression patterns for the genes in one G-box, one MYB, and one W-box motif. These genes are regulated in plants upon pathogen treatments. Data according to AtGenExpress.
(PDF)

Table S1 The 120,276 co-expressed gene pairs in the GGM network.
(XLSX)

Table S2 The results of motif enrichment and motif position bias analysis for the 4 analyzed motifs.
(XLSX)

Table S3 The gene lists for the modules identified in Figure 2 to Figure 8.
(XLSX)

Table S4 The gene co-expression modules regulated by the 4 analyzed motifs in the AGCN network.
(XLSX)

Table S5 The treatments used in the gene regulation profiling experiment in Figure S1, S2, S6, S17, and S18.
(XLSX)

Table S6 The tissues used in the expression profiling experiments in Figure S3 and S7.
(XLSX)
