# Supplementary figures and images for: Incorporating Motif Analysis into Gene Co-expression Networks Reveals Novel Modular Expression Pattern and New Signaling Pathways
Source: PLoS Genet. 2013 Oct 3;9(10):e1003840. doi: 10.1371/journal.pgen.1003840 (PMC3789834; doi:10.1371/journal.pgen.1003840)

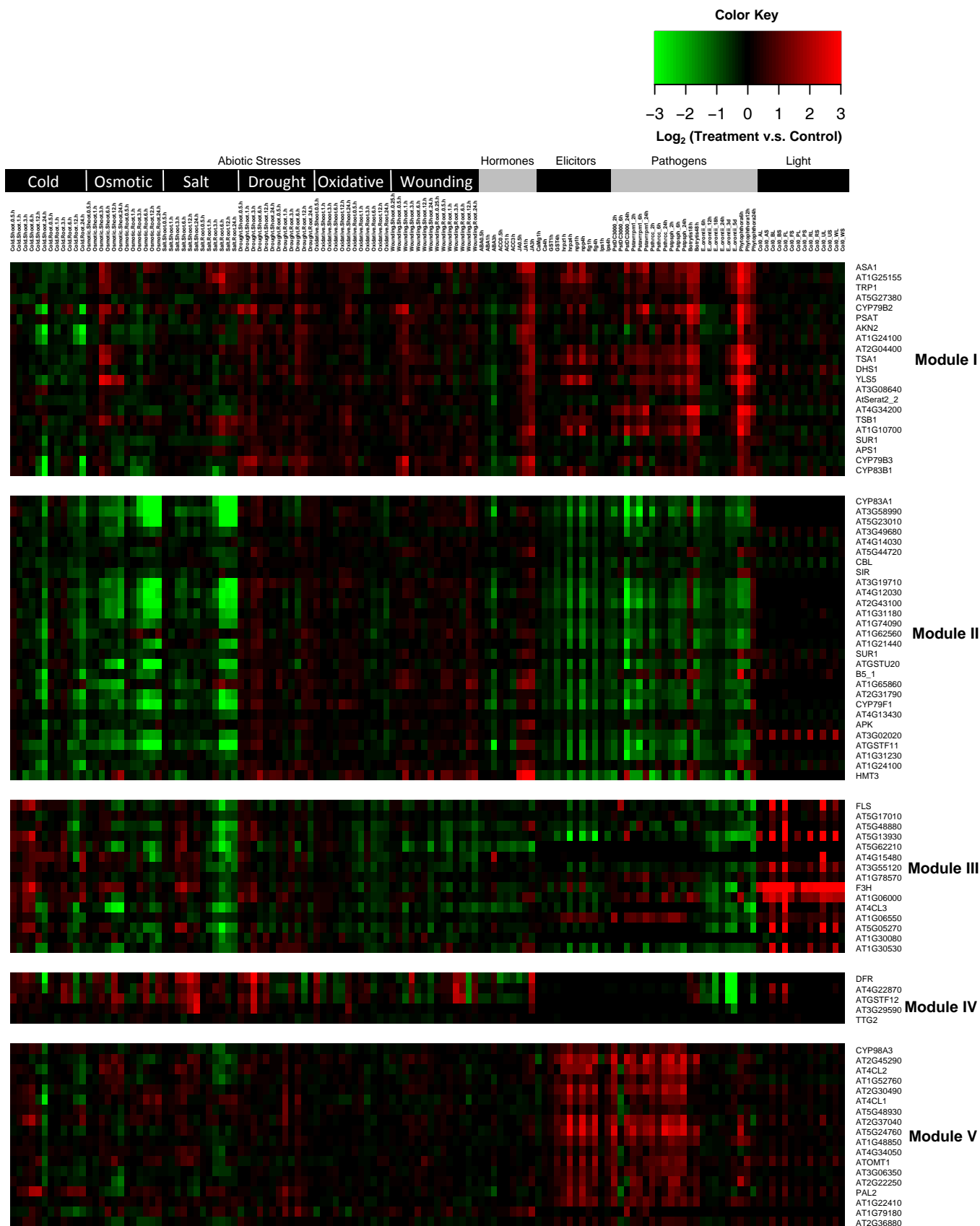

**Figure S2**  
**Ma et al.**

Supplement: Figure S2 — Expression pattern for genes in five MYB modules after different treatments. Data according to AtGenExpress. (PDF) [file pgen.1003840.s002.pdf]

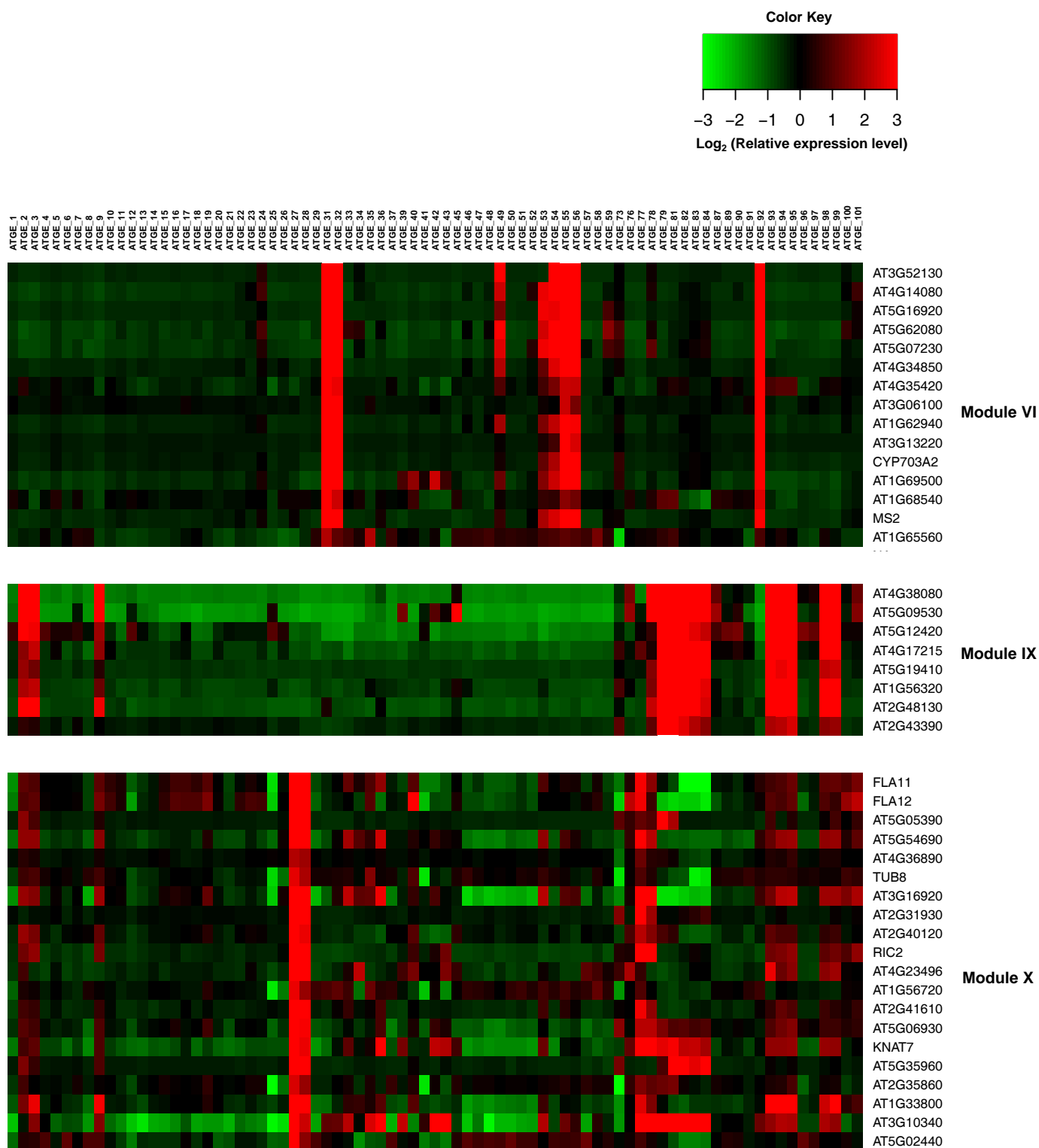

**Figure S3**  
**Ma et al.**

Supplement: Figure S3 — Expression pattern for the genes in three MYB modules in different tissues. Data are represented as relative expression levels. Data according to AtGenExpress. (PDF) [file pgen.1003840.s003.pdf]

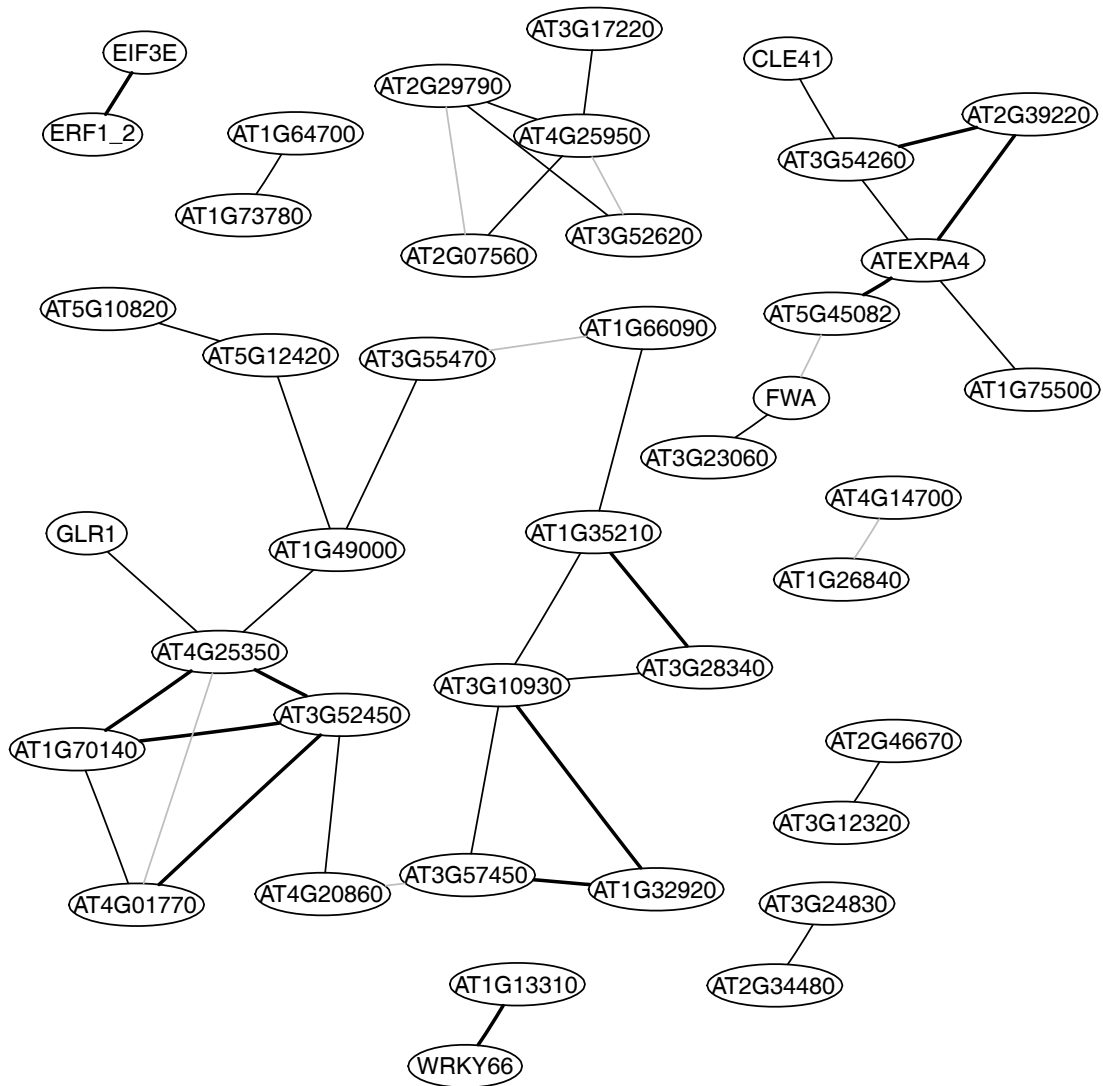

**Figure S4**  
Ma et al.

Supplement: Figure S4 — A typical sub-network for the genes recovered for MYB with pValue< = 0.01 in a permutation expression. Three modules with > = 5 genes were identified. The solid and grey lines indicate gene pairs with top 20% or bottom 20% partial correlation values respectively. (PDF) [file pgen.1003840.s004.pdf]

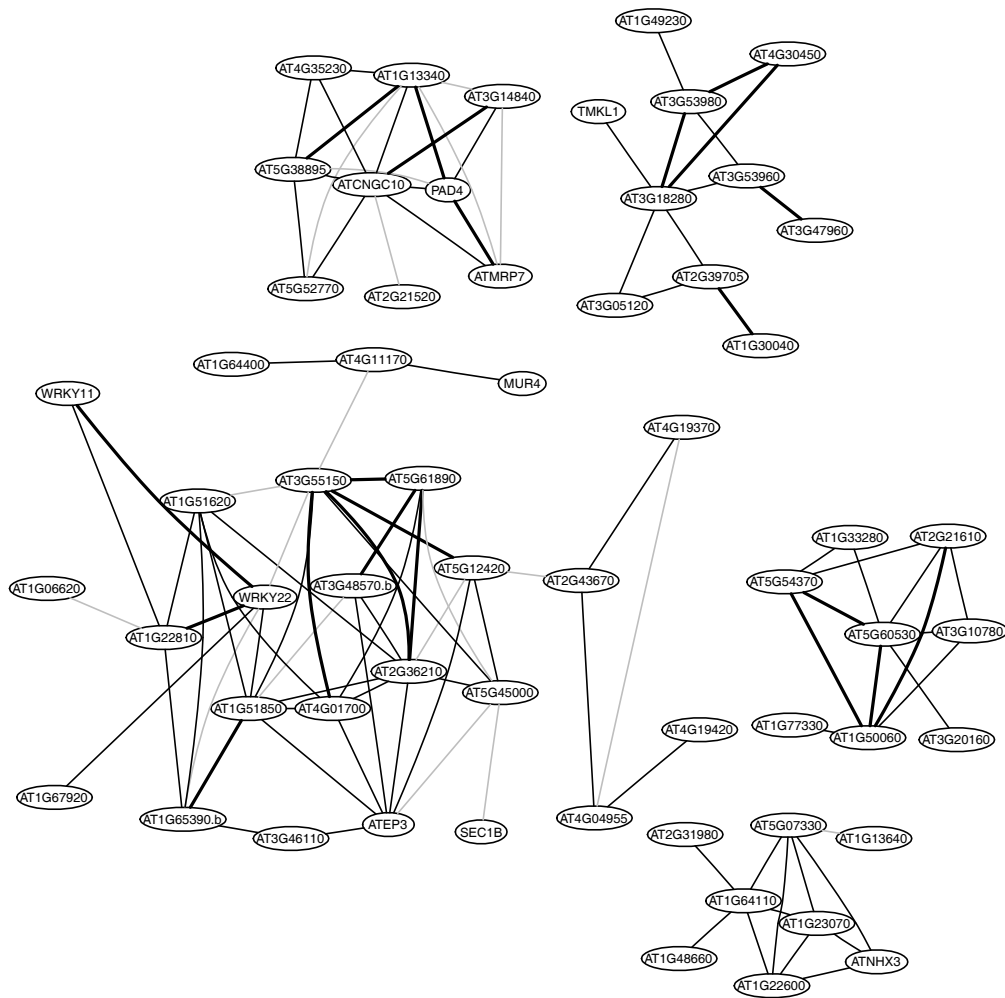

**Figure S5**  
Ma et al.

Supplement: Figure S5 — A typical sub-network for the genes recovered for MYB with z-score> = 2.2 in a permutation expression. Five modules with > = 5 genes were identified. The solid and grey lines indicate gene pairs with high or low partial correlation values respectively. (PDF) [file pgen.1003840.s005.pdf]

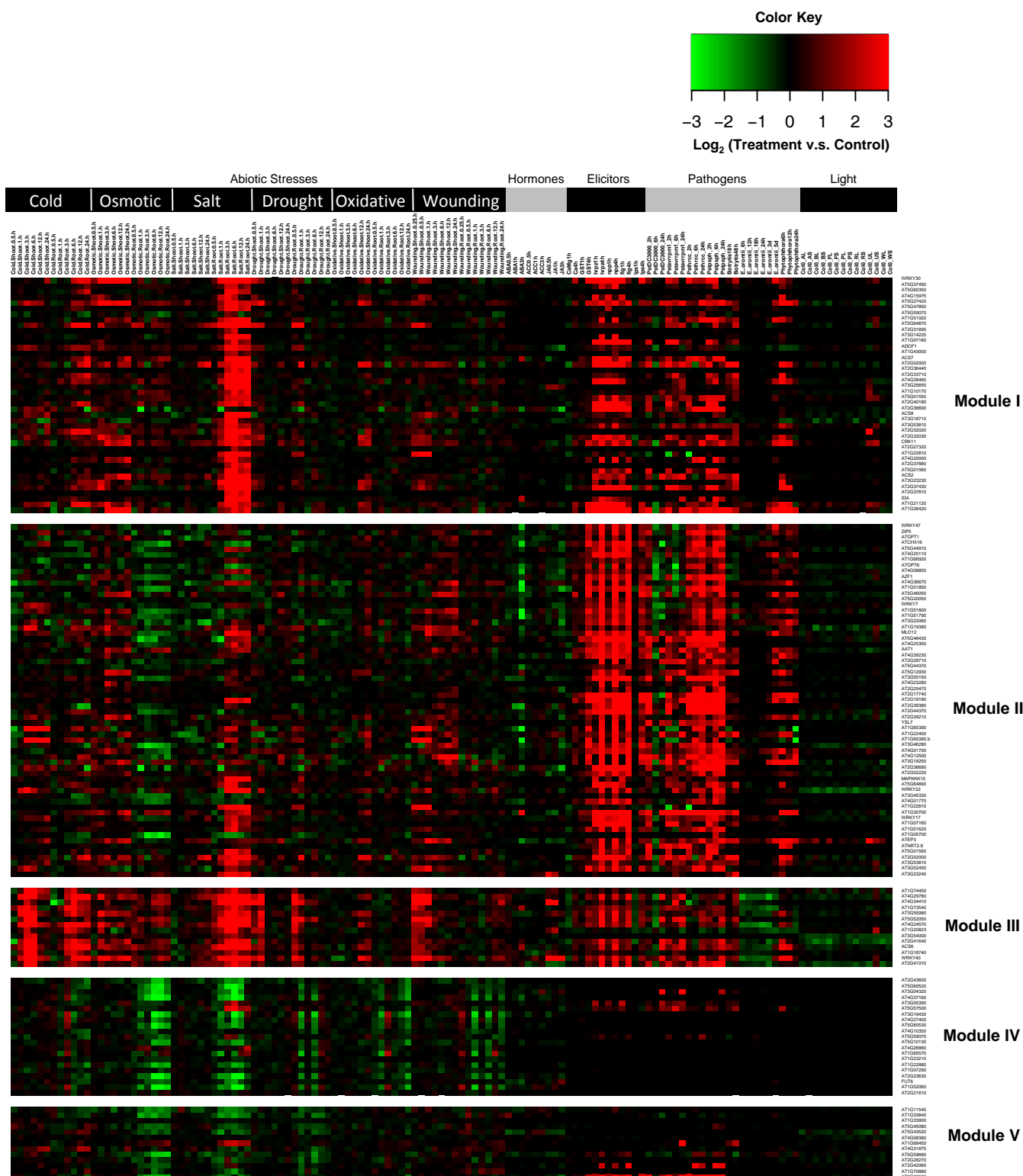

**Figure S6**  
**Ma et al.**

Supplement: Figure S6 — Expression patterns for the genes in five W-box modules upon different treatments. Data according to AtGenExpress. (PDF) [file pgen.1003840.s006.pdf]

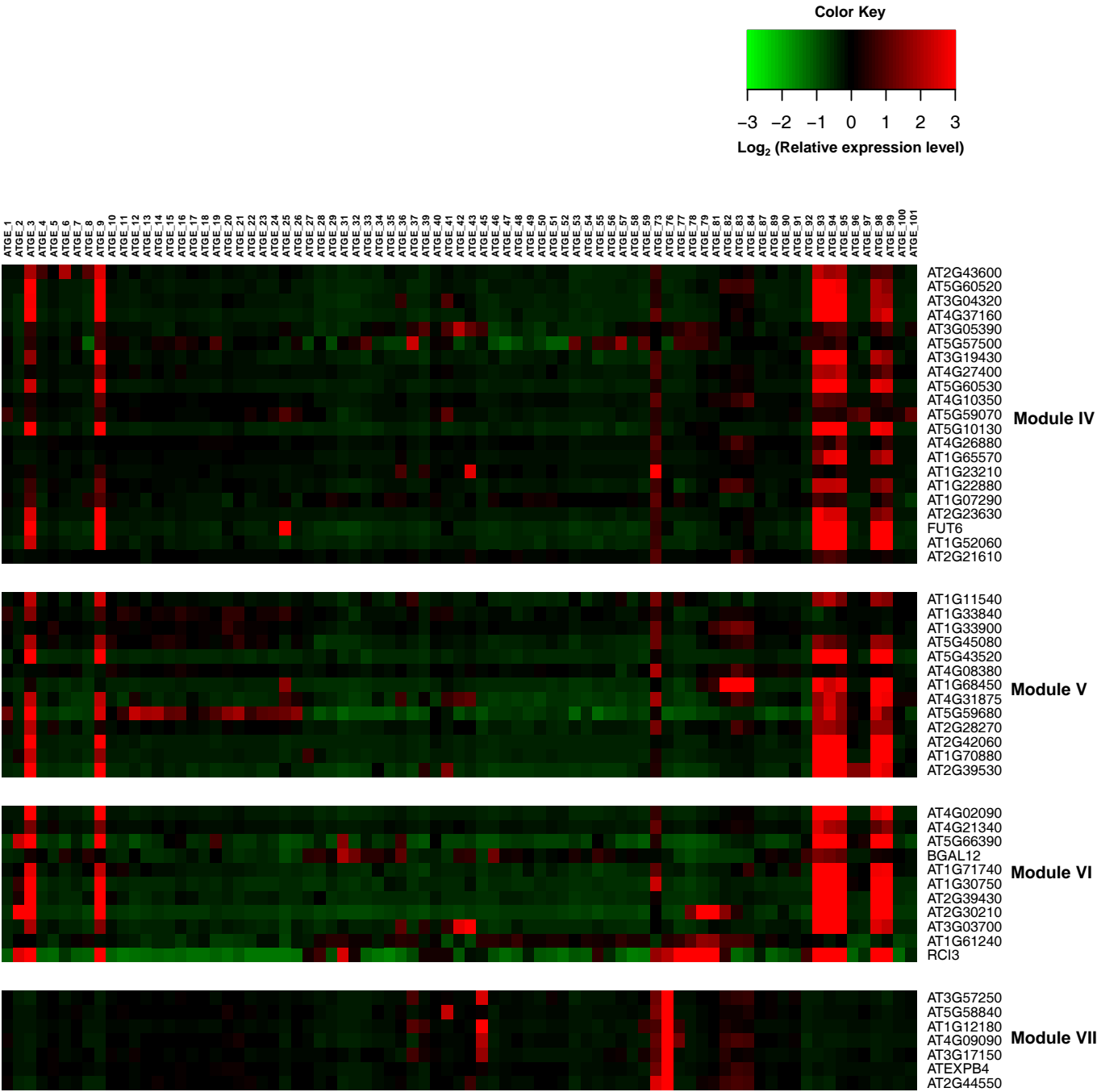

**Figure S7**  
**Ma et al.**

Supplement: Figure S7 — Expression patterns for the genes in two W-box modules in different tissues. Data are represented as relative expression levels. Data according to AtGenExpress. (PDF) [file pgen.1003840.s007.pdf]

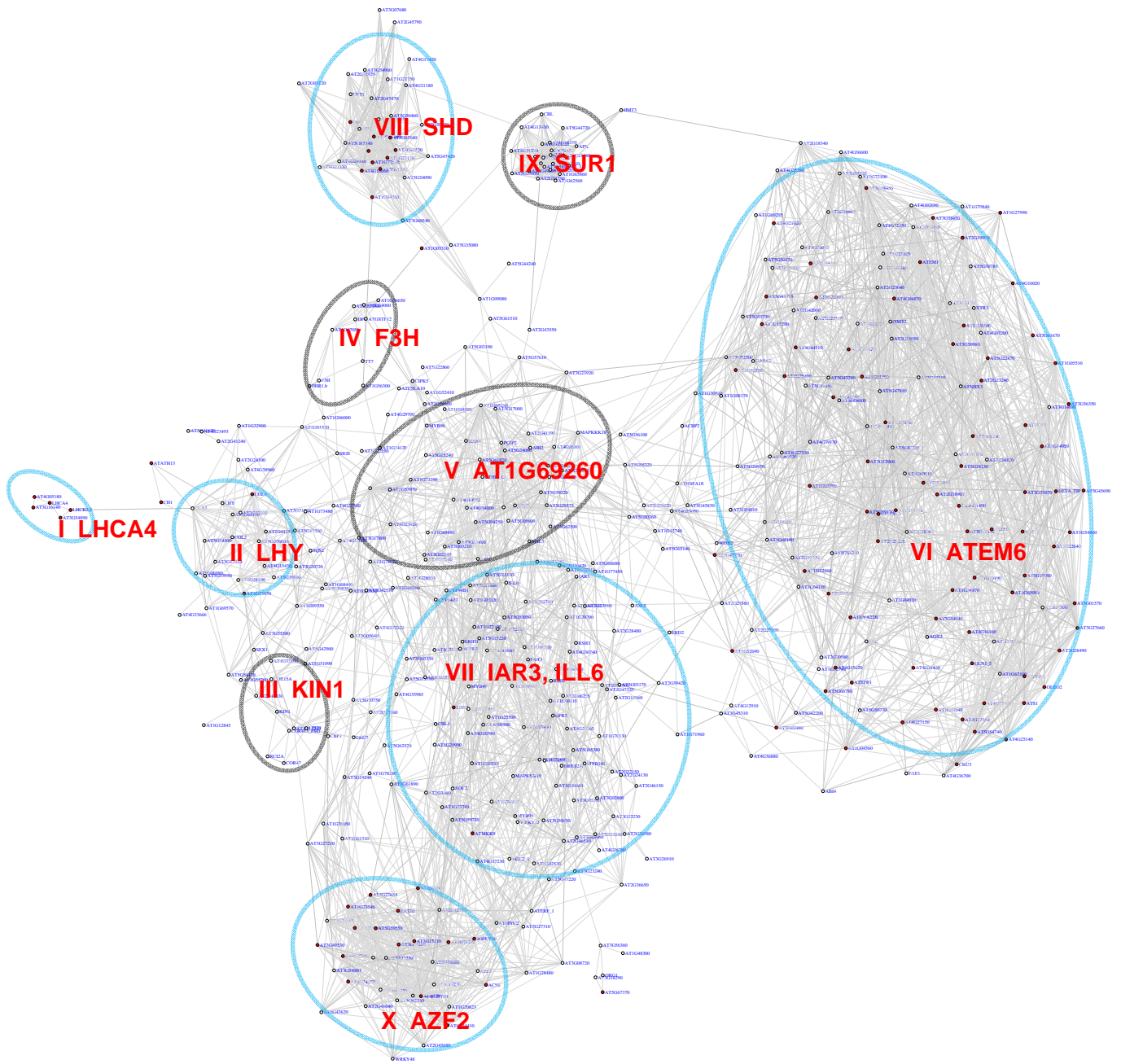

**Figure S8**  
**Ma et al.**

Supplement: Figure S8 — Comparison between the GGM network (bottom-up approach) and the AGCN network (top-down approach). The sub-network identified for the G-box motif via motif enrichment analysis for the GGM network shown in Figure 2 is intersected with the G-box modules identified in the AGCN network. Red nodes - genes identified in both methods; white nodes – genes identified only in the GGM network. Circled in grey are modules identified only via the GGM methods. Modules identified in both methods are circled in blue. (PDF) [file pgen.1003840.s008.pdf]

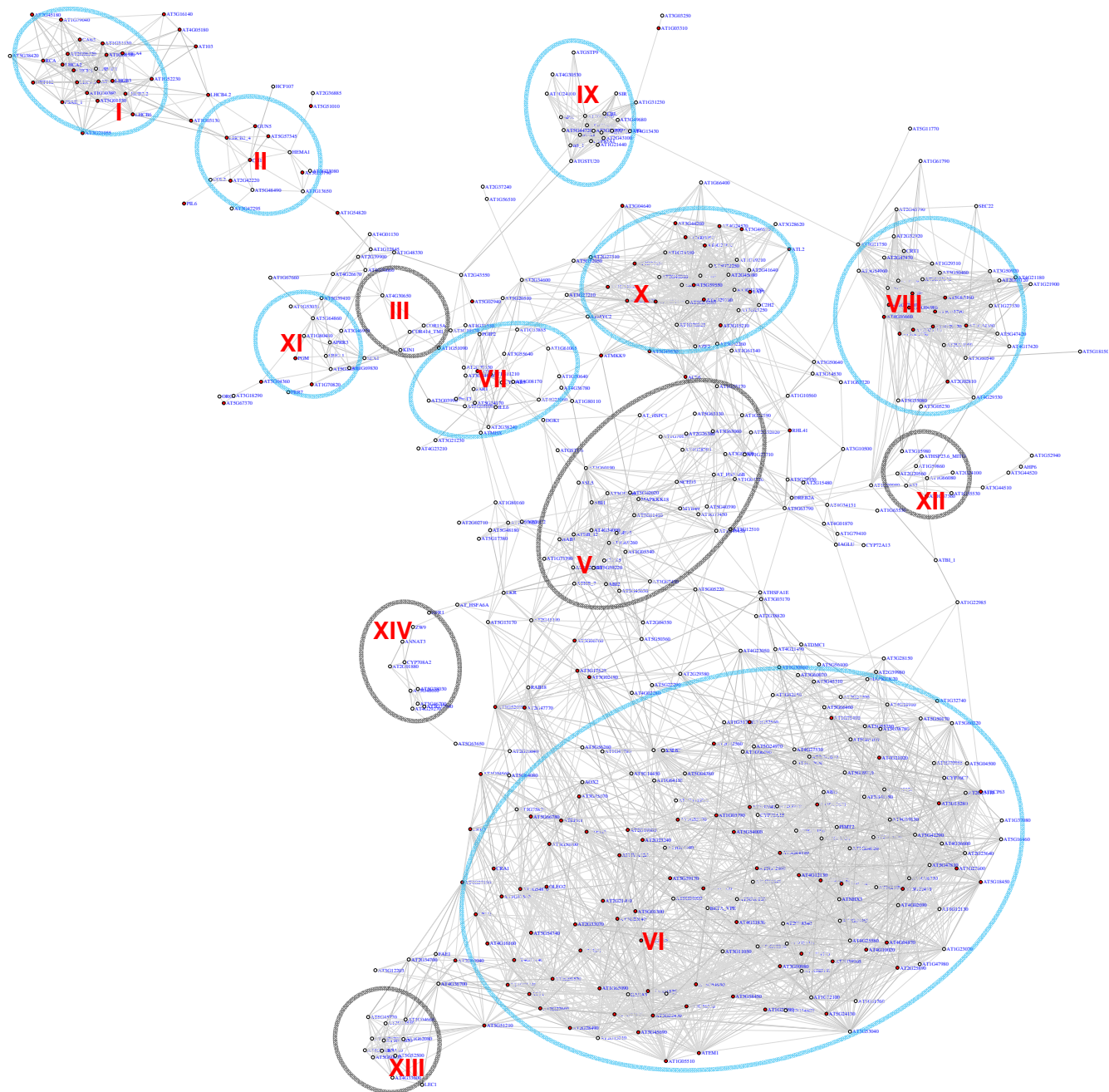

**Figure S9**  
**Ma et al.**

Supplement: Figure S9 — The sub-network identified for the G-box motif via position bias analysis for the GGM network shown in Figure 3 is intersected with the G-box modules identified in the AGCN network. Red nodes - genes identified in both methods; white nodes – genes identified only in the GGM network. Circled in grey are modules identified only via the GGM methods. Modules identified in both methods are circled in blue. (PDF) [file pgen.1003840.s009.pdf]

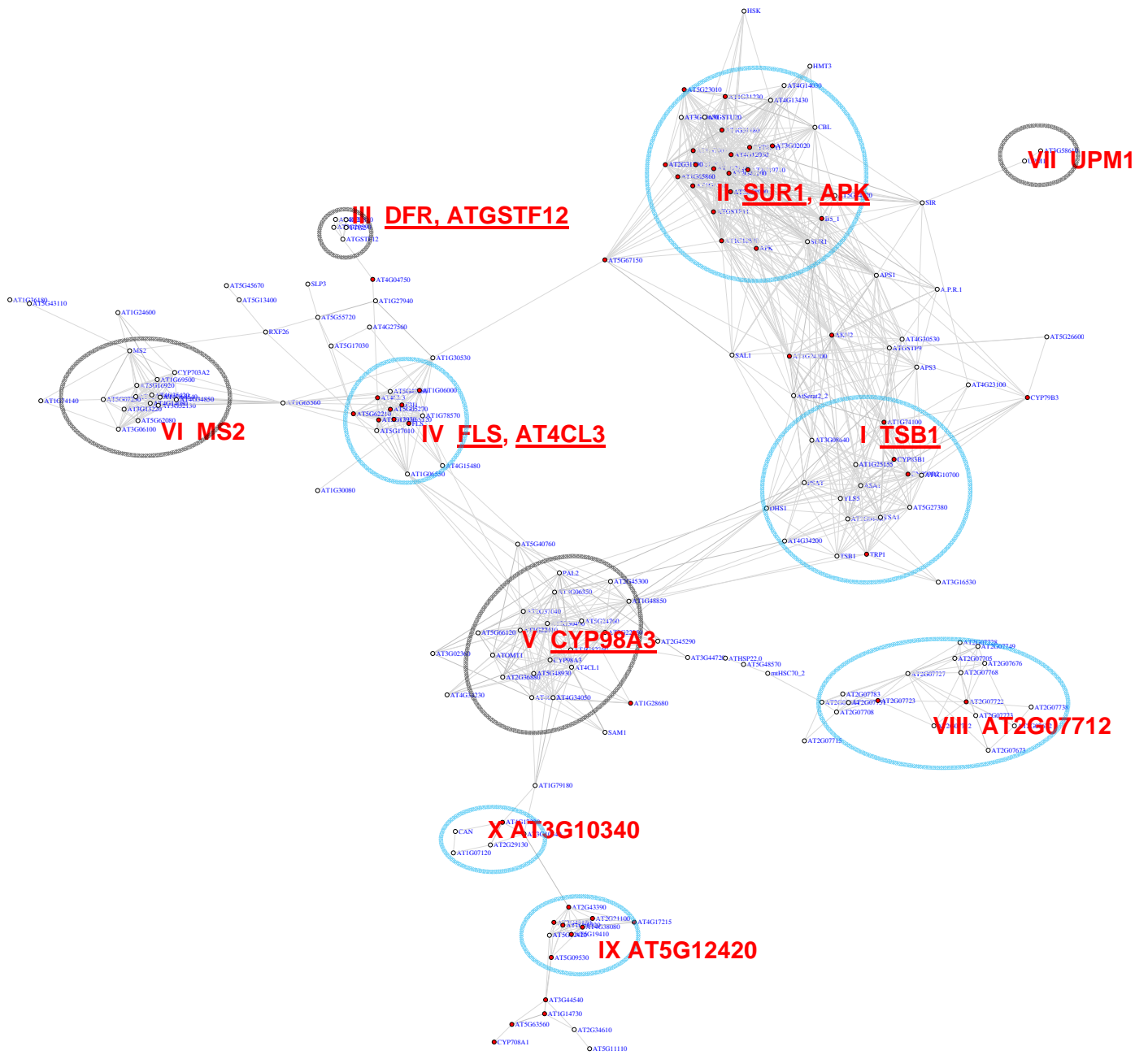

Figure S10  
Ma et al.

Supplement: Figure S10 — The sub-network identified for the MYB motif via motif enrichment analysis for the GGM network shown in Figure 4 is intersected with the MYB modules identified in the AGCN network. Red nodes - genes identified in both methods; white nodes – genes identified only in the GGM network. Circled in grey are modules identified only via the GGM methods. Modules identified in both methods are circled in blue. (PDF) [file pgen.1003840.s010.pdf]

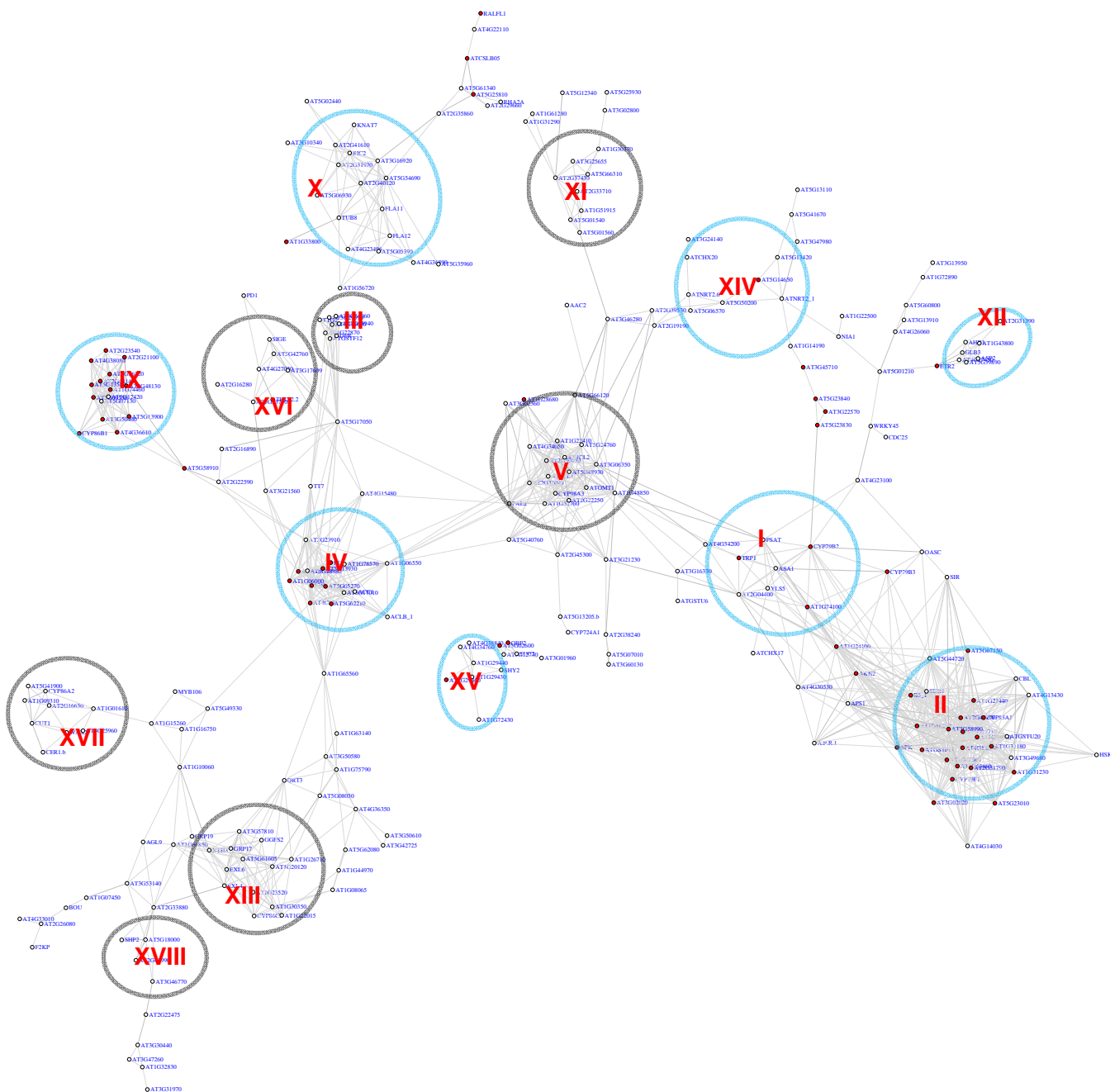

**Figure S11**  
**Ma et al.**

Supplement: Figure S11 — The sub-network identified for the MYB motif via position bias analysis for the GGM network shown in Figure 5 is intersected with the MYB modules identified in the AGCN network. Red nodes - genes identified in both methods; white nodes genes identified only in the GGM network. Circled in grey are modules identified only via the GGM methods. Modules identified in both methods are circled in blue. (PDF) [file pgen.1003840.s011.pdf]

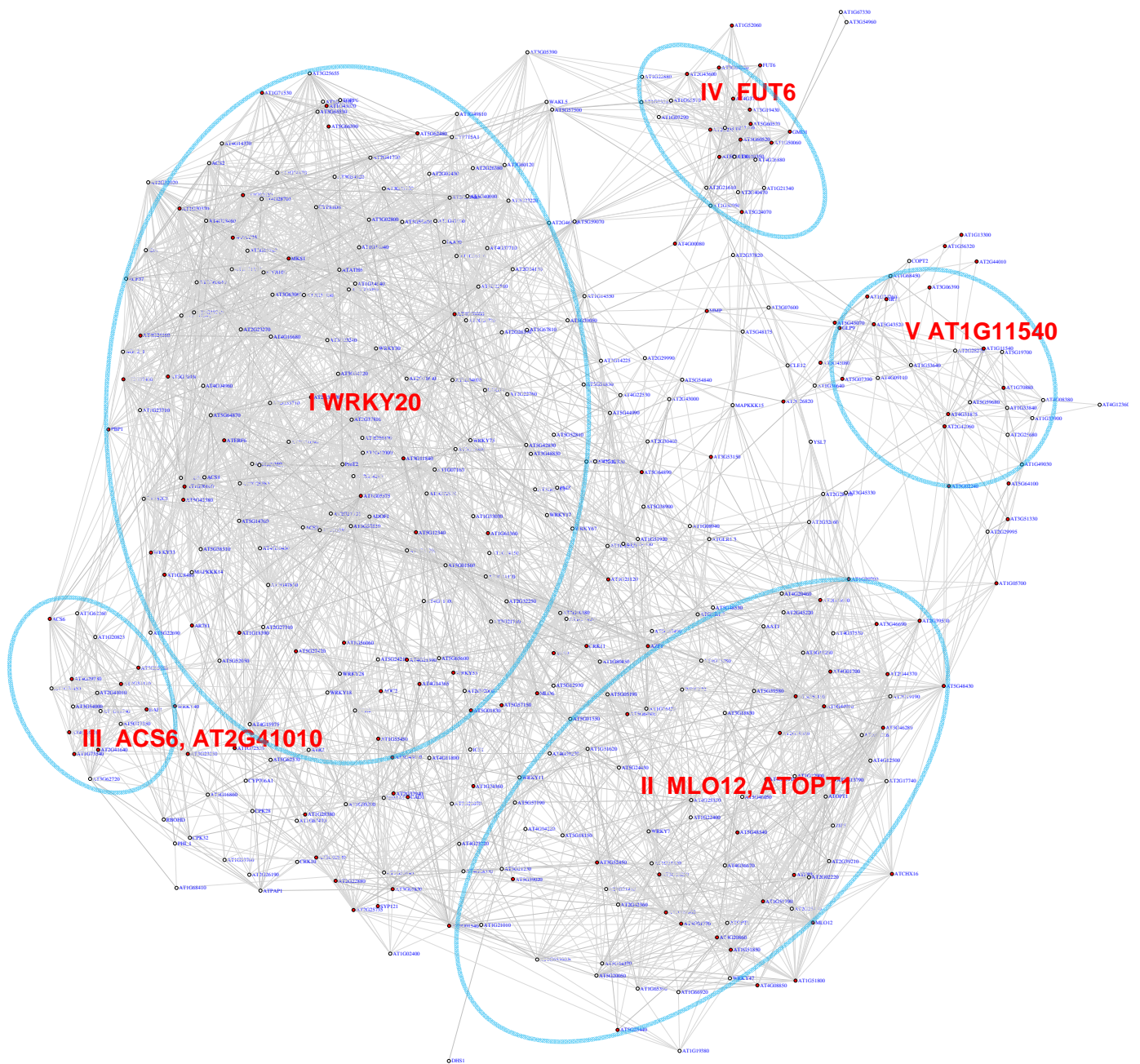

**Figure S12**  
**Ma et al.**

Supplement: Figure S12 — The sub-network identified for the W-box motif via motif enrichment analysis for the GGM network shown in Figure 6 is intersected with the W-box modules identified in the AGCN network. Red nodes - genes identified in both methods; white nodes – genes identified only in the GGM network. Modules identified in both methods are circled in blue. (PDF) [file pgen.1003840.s012.pdf]

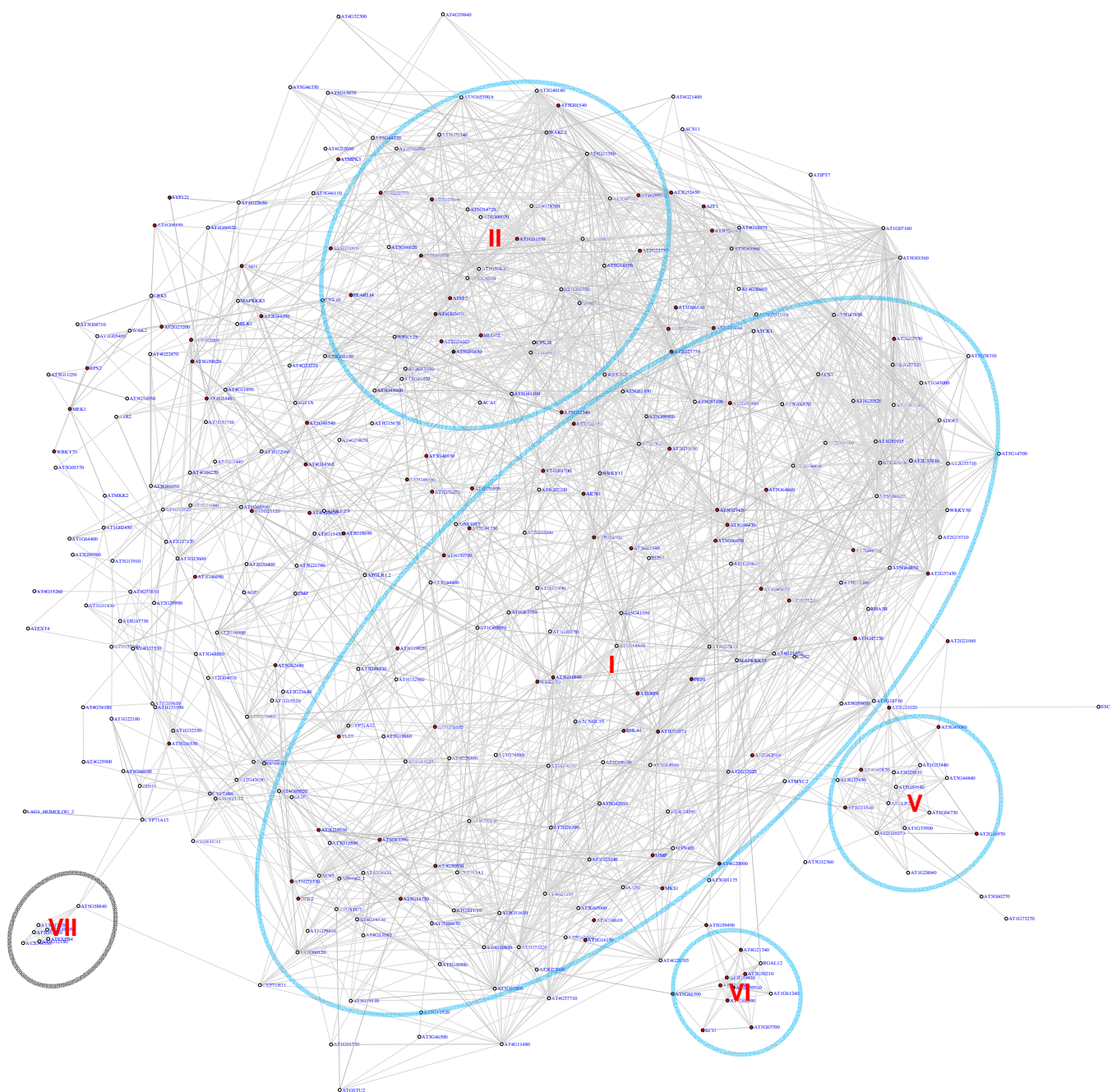

**Figure S13**  
**Ma et al.**

Supplement: Figure S13 — The sub-network identified for the W-box motif via position bias analysis for the GGM network shown in Figure 7 is intersected with the MYB modules identified in the AGCN network. Red nodes - genes identified in both methods; white nodes – genes identified only in the GGM network. Circled in grey are modules identified only via the GGM methods. Modules identified in both methods are circled in blue. (PDF) [file pgen.1003840.s013.pdf]

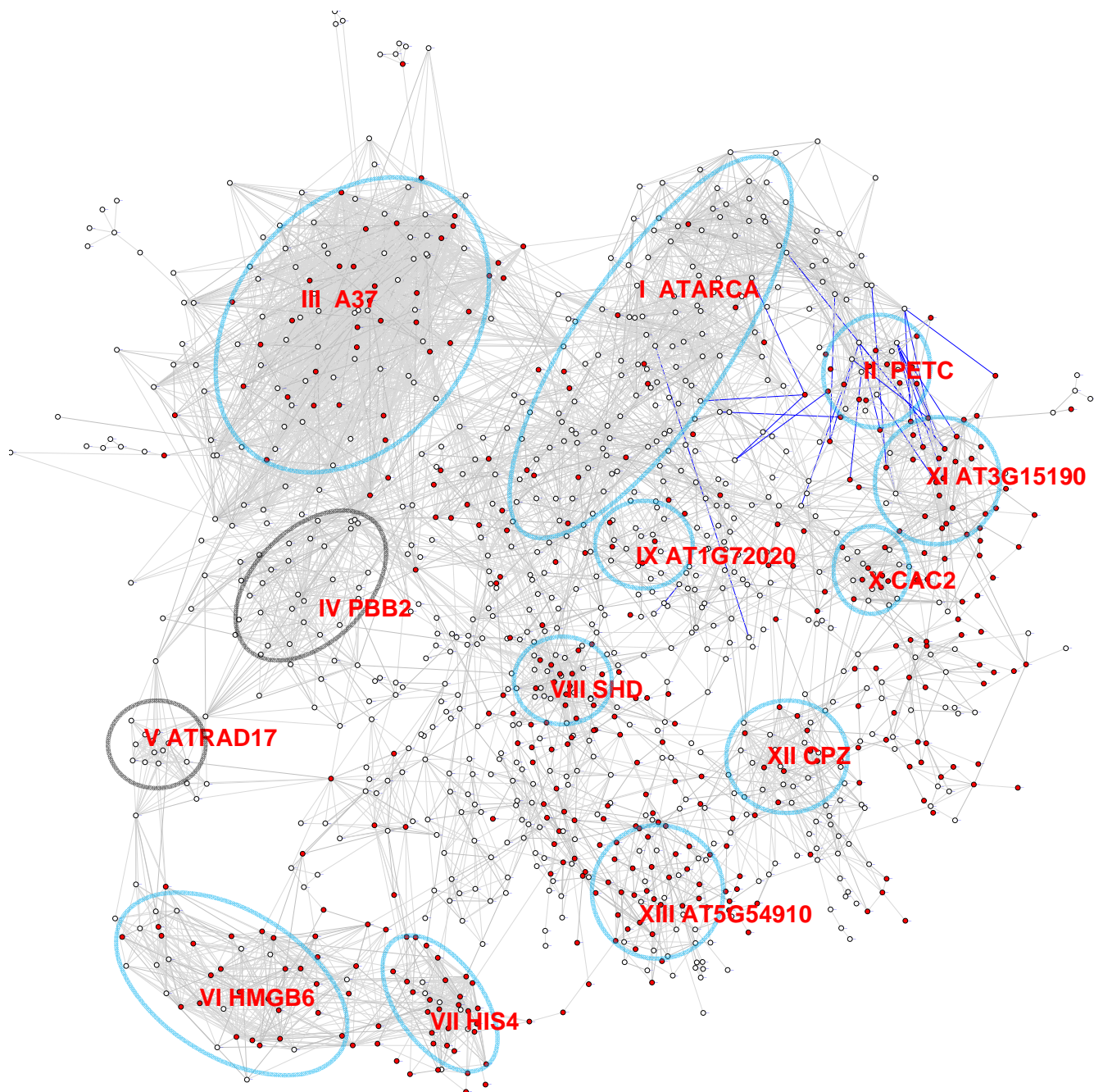

**Figure S14**  
Ma et al.

Supplement: Figure S14 — The sub-network identified for the site II element motif via position bias analysis for the GGM network shown in Figure 8 is intersected with the site II element modules identified in the AGCN network. Red nodes - genes identified in both methods; white nodes – genes identified only in the GGM network. Circled in grey are modules identified only via the GGM methods. Modules identified in both methods are circled in blue. Blue lines connecting two genes indicated that they have negative correlated expression pattern. (PDF) [file pgen.1003840.s014.pdf]

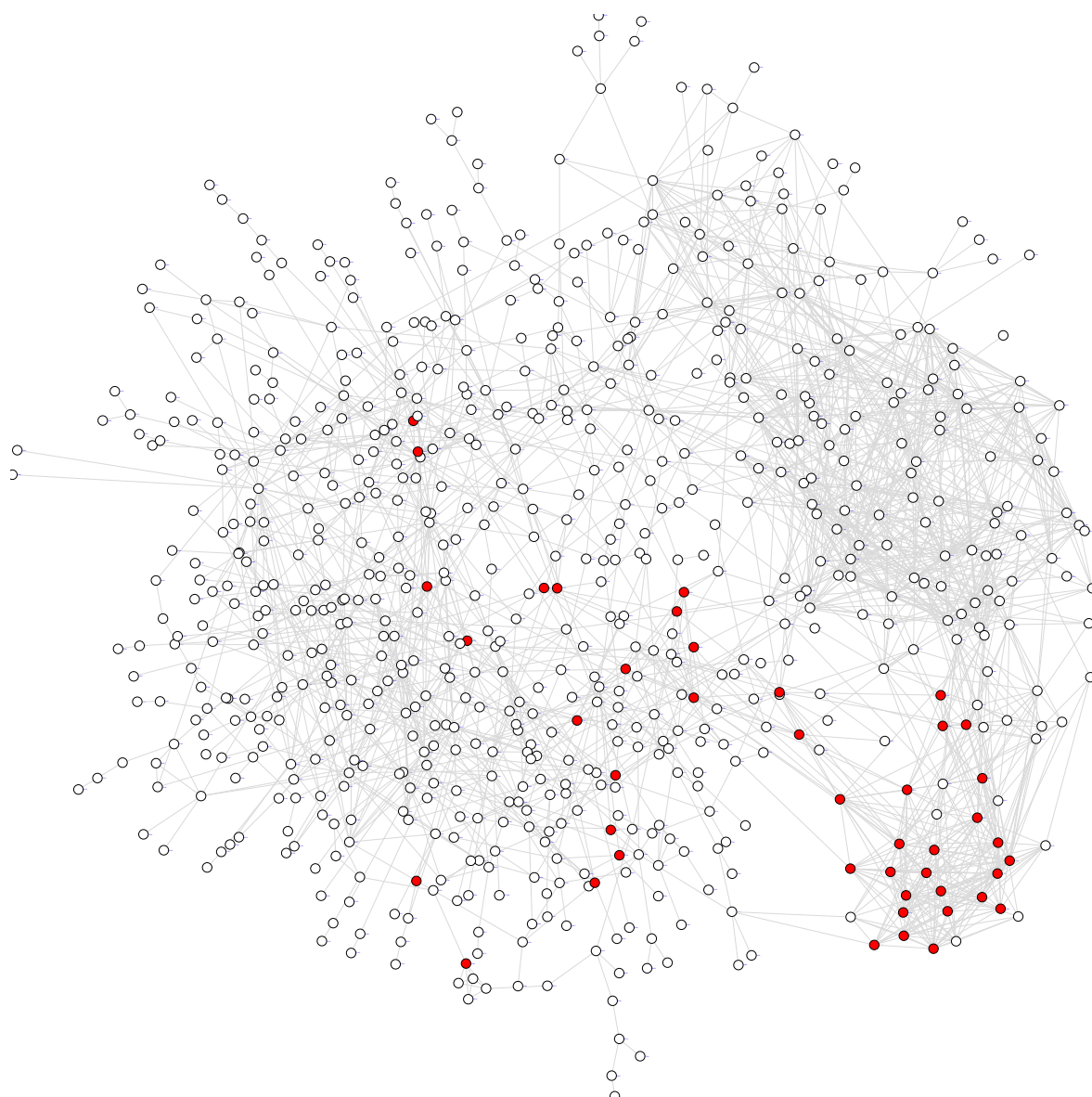

**Figure S15**  
**Ma et al.**

Supplement: Figure S15 — A sub-network extracted for the 1,362 genes in the AGCN cluster No. 1 from the GGM network. Labeled in red are those genes deemed to be regulated by the G-box motif via our bottom-up approach analysis on the GGM network. (PDF) [file pgen.1003840.s015.pdf]

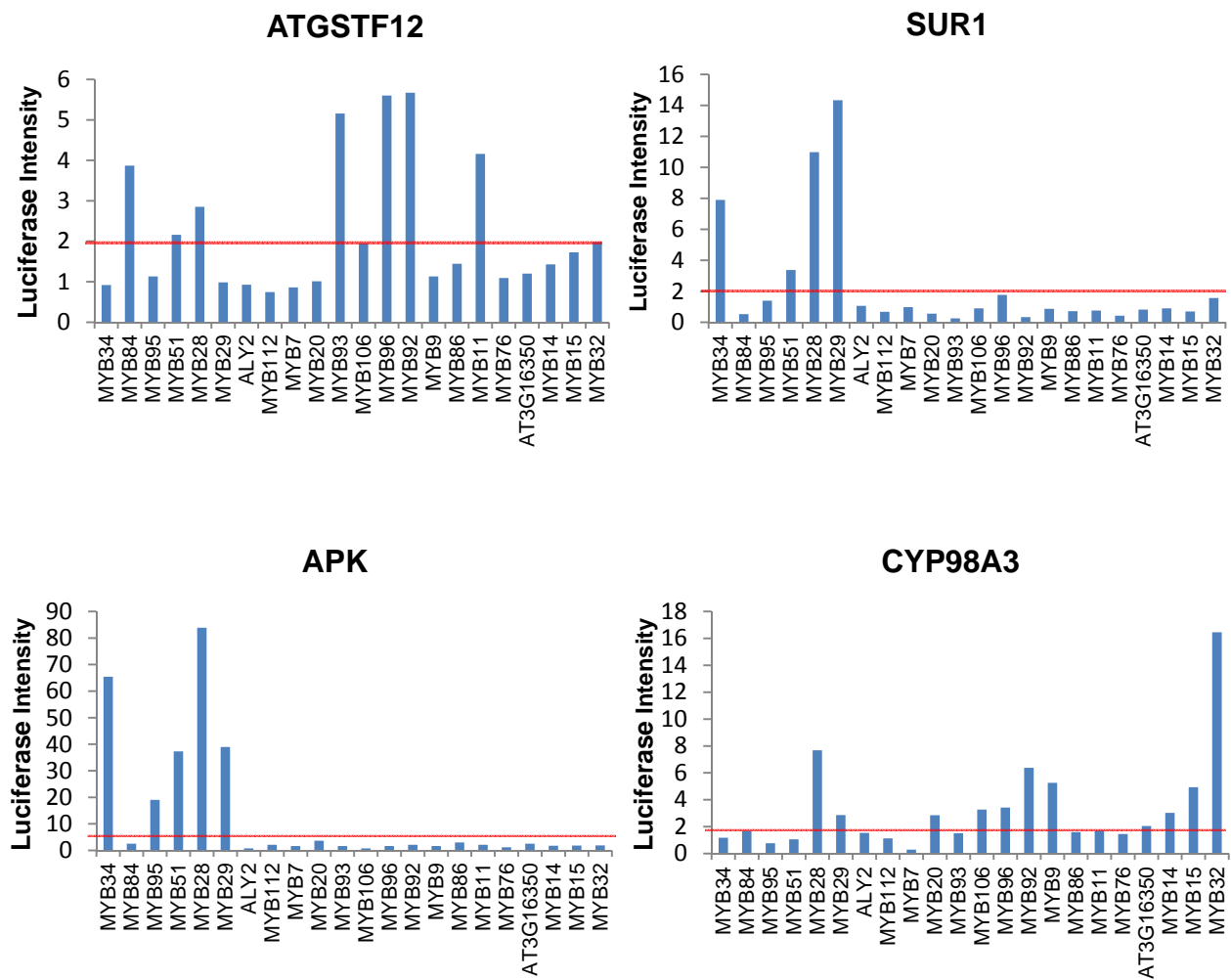

**Figure S16**  
Ma et al.

Supplement: Figure S16 — The interactions between MYBs and selected promoters. Assays conducted with the dual luciferase system. Plotted are the relative luciferase activities for different MYB plus Promoter::LUC combination. The red lines in each panel indicate the threshold level for interaction. Name on the top of the graph indicates promoter of the gene used in the assay. Different MYBs used are shown below each bar on the X-axis. (PDF) [file pgen.1003840.s016.pdf]

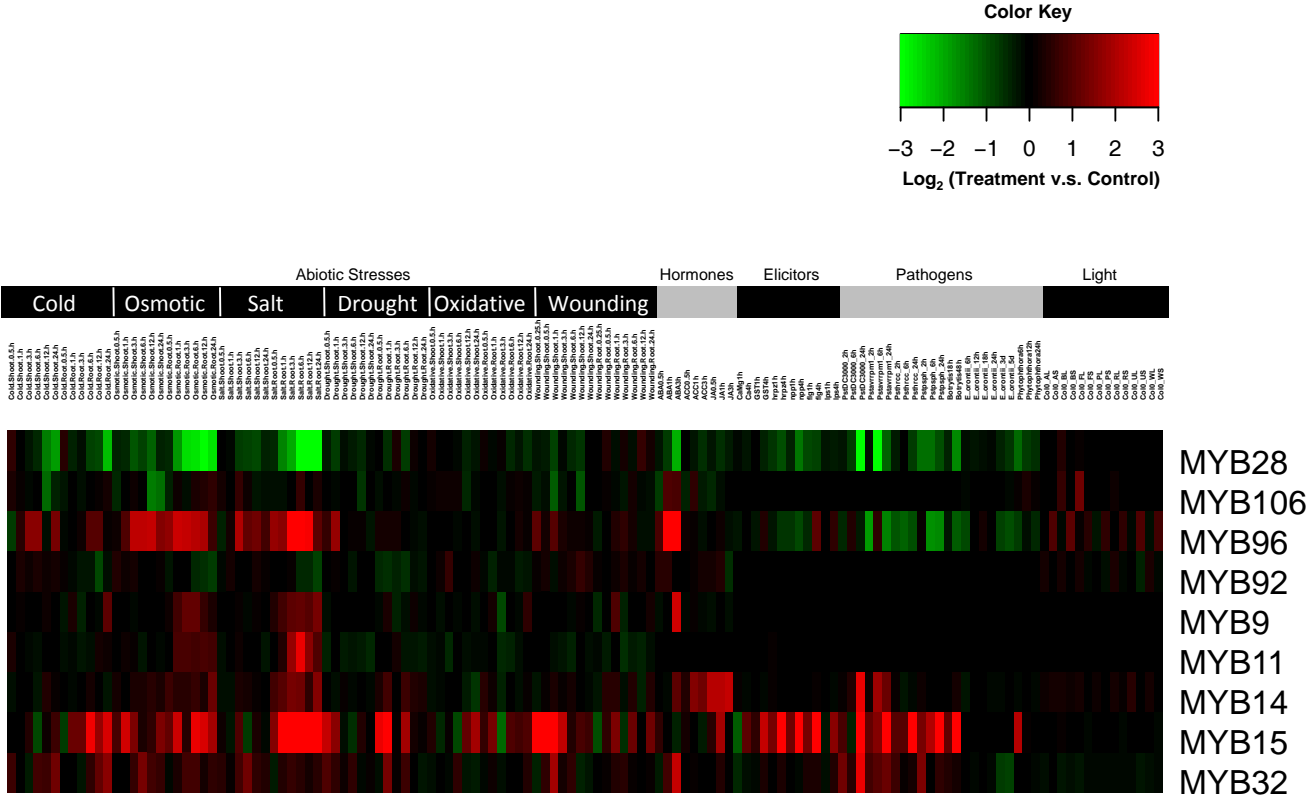

Figure S17  
Ma et al.

Supplement: Figure S17 — Expression patterns for different MYB TFs under different treatments. Data according to AtGenExpress. (PDF) [file pgen.1003840.s017.pdf]
