# Supplementary material for: Incorporating Motif Analysis into Gene Co-expression Networks Reveals Novel Modular Expression Pattern and New Signaling Pathways
Source: PLoS Genet. 2013 Oct 3;9(10):e1003840. doi: 10.1371/journal.pgen.1003840 (PMC3789834; doi:10.1371/journal.pgen.1003840)
Supplement: Figure S1 — Expression pattern for genes in G-box modules after different treatments. Data according to AtGenExpress. (PDF) [file pgen.1003840.s001.pdf]

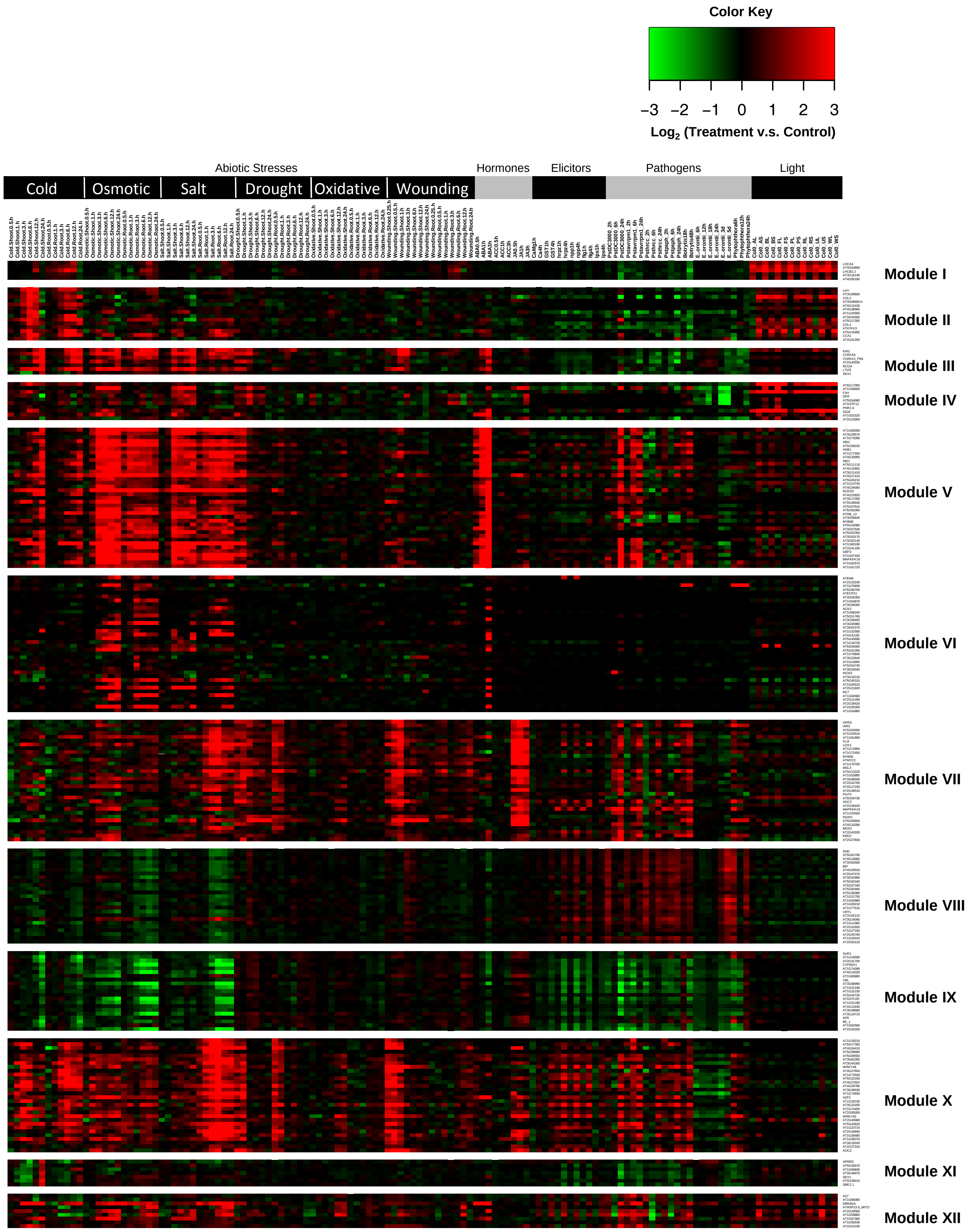

Figure S1
Ma et al.
