# Supplementary material for: Incorporating Motif Analysis into Gene Co-expression Networks Reveals Novel Modular Expression Pattern and New Signaling Pathways
Source: PLoS Genet. 2013 Oct 3;9(10):e1003840. doi: 10.1371/journal.pgen.1003840 (PMC3789834; doi:10.1371/journal.pgen.1003840)
Supplement: Figure S18 — Expression patterns for the genes in one G-box, one MYB, and one W-box motif. These genes are regulated in plants upon pathogen treatments. Data according to AtGenExpress. (PDF) [file pgen.1003840.s018.pdf]

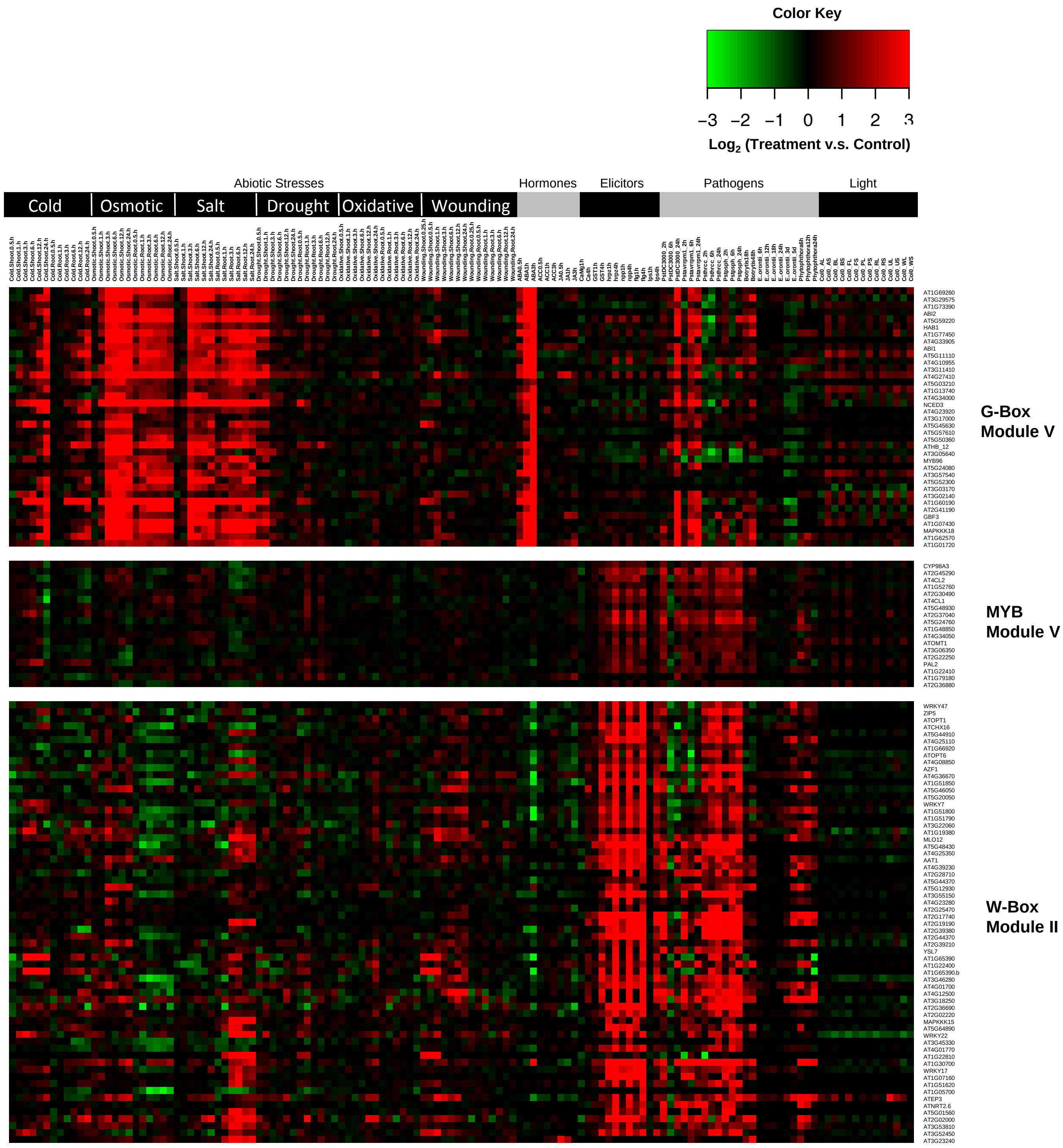

Figure S18
Ma et al.
